# Increased apical Na^+^ permeability in cystic fibrosis is supported by a quantitative model of epithelial ion transport

**DOI:** 10.1113/jphysiol.2013.253955

**Published:** 2013-06-03

**Authors:** Donal L O’Donoghue, Vivek Dua, Guy W J Moss, Paola Vergani

**Affiliations:** 1 Centre for Mathematics and Physics in the Life Sciences and Experimental Biology, University College London Gower Street, London, WC1E 6BT, United Kingdom; 2 Department of Chemical Engineering, Centre for Process Systems Engineering, University College London Torrington Place, London, WC1E 7JE, United Kingdom; 3 Department of Neuroscience, Physiology & Pharmacology, University College London Gower Street, London, WC1E 6BT, United Kingdom

## Abstract

Cystic fibrosis (CF) is caused by mutations in the cystic fibrosis transmembrane conductance regulator (CFTR) gene, which encodes an anion channel. In the human lung CFTR loss causes abnormal ion transport across airway epithelial cells. As a result CF individuals produce thick mucus, suffer persistent bacterial infections and have a much reduced life expectancy. Trans-epithelial potential difference (*V*_t_) measurements are routinely carried out on nasal epithelia of CF patients in the clinic. CF epithelia exhibit a hyperpolarised basal *V*_t_ and a larger *V*_t_ change in response to amiloride (a blocker of the epithelial Na^+^ channel, ENaC). Are these altered bioelectric properties solely a result of electrical coupling between the ENaC and CFTR currents, or are they due to an increased ENaC permeability associated with CFTR loss? To examine these issues we have developed a quantitative mathematical model of human nasal epithelial ion transport. We find that while the loss of CFTR permeability hyperpolarises *V*_t_ and also increases amiloride-sensitive *V*_t_, these effects are too small to account for the magnitude of change observed in CF epithelia. Instead, a parallel increase in ENaC permeability is required to adequately fit observed experimental data. Our study provides quantitative predictions for the complex relationships between ionic permeabilities and nasal *V*_t_, giving insights into the physiology of CF disease that have important implications for CF therapy.

**Key points**

- Cystic fibrosis (CF) is a common genetic disease caused by loss-of-function mutations in the cystic fibrosis transmembrane conductance regulator gene, which encodes a channel protein, selective for anions.
- In the lungs, the site of the most severe symptoms, CF causes abnormal electrolyte transport in epithelial cells which line the airways.
- Airway epithelial ion transport can be assessed by measuring the trans-epithelial potential difference (*V*_t_) which shows characteristic changes in CF individuals. We developed a biophysical model of ion transport in human nasal epithelia, in order to investigate quantitatively which transport parameters underlie these observed bioelectric changes.
- We found that loss of apical Cl^−^ permeability alone is insufficient to explain the bioelectric properties of CF epithelia. An increase of apical Na^+^ permeability must also occur.
- This insight has important implications for our understanding of the physiology of CF disease, and hence for potential therapies aimed at correcting the CF ion transport defect.

## Introduction

Cystic fibrosis (CF) is a mono-genetic disorder that impairs quality of life and greatly reduces life expectancy (Davies *et al.* 2007). It is the most common fatal inherited genetic disease found in people of European descent (Dodge *et al.* 2007). CF is a complex disease, affecting several organs; however, the most frequent cause of death amongst CF sufferers is lung failure resulting from persistent bacterial infections.

It is known that loss-of-function mutations in the *CFTR* gene product, an anion-selective channel, are the root cause of the disease. Thus, abnormal trans-epithelial electrolyte transport appears to be crucial to the pathogenesis of CF (Rowe *et al.* 2005). Measurements of the trans-epithelial potential difference (*V*_t_) across nasal epithelia can be used to investigate airway epithelial ion transport and such measurements are often made *in vivo* to aid diagnosis of CF in the clinic. *V*_t_ measurements are also used as outcome measures in clinical trials of drug and gene therapies for the disease (Rowe *et al.* 2011). CF epithelia show hyperpolarised basal *V*_t_ (relative to non-CF epithelia), an increased depolarisation following block of the epithelial Na^+^ channel (ENaC) with its inhibitor amiloride, a reduced or missing response when the driving force for apical Cl^−^ efflux is increased, and no hyperpolarisation in response to raised intracellular cAMP levels (Knowles *et al.* 1995).

These bioelectric properties arise as a direct result of mutations in the *CFTR* gene, but whether or not they are simply a consequence of the loss of apical anion permeability is a matter of debate. It has been suggested that CFTR regulates the activity of other transport processes in epithelial cells, in particular ENaC, with the loss of CFTR resulting in higher basal levels of apical Na^+^ conductance (Stutts *et al.* 1995; Donaldson & Boucher, 2007). More recent studies, however, report that Na^+^ absorption in pig and human CF airway epithelial cultures is not increased (Chen *et al.* 2010; Itani *et al.* 2011). These studies suggest that the loss of anion conductance can account for hyperpolarised basal *V*_t_, as well as the increased amiloride-sensitive *V*_t_ and altered short-circuit current, because of the way the CFTR currents are electrically coupled to other transport processes. Previous modelling work from a kidney epithelial cell line provides qualitative support for this idea (Horisberger, 2003).

To assess these conflicting views we developed a detailed mathematical model of ion transport in human nasal epithelial (HNE) cells, so as to quantitatively investigate the relationship between individual ionic permeabilities and commonly measured bioelectric properties of the integrated epithelial transport system, such as basal *V*_t_ and amiloride-sensitive *V*_t_. Our model differs from most previous studies investigating airway epithelial physiology (Hartmann & Verkman, 1990; Duszyk & French, 1991; Warren *et al.* 2009; Falkenberg & Jakobsson, 2010) in that it focuses specifically on nasal epithelial cell components and parameter values. A modelling study focused on quantifying ionic permeabilities in non-CF HNE cells has recently been published, but this work did not consider ion transport in CF (Garcia *et al.* 2013). We use data from primary cultures of both CF and non-CF nasal epithelial cells for model validation, thus allowing us to investigate clinically relevant questions regarding how changes in the underlying transport components give rise to altered nasal *V*_t_ measurements in CF.

We found that while the electrical coupling between CFTR and ENaC currents can cause, qualitatively, the type of changes seen in CF, the magnitudes of these effects are not large enough to explain CF abnormalities. Instead, apical Na^+^ permeability must be increased in CF in order to quantitatively explain the differences observed in bioelectric properties between the non-CF and CF airway epithelium.

## Methods

### Model overview

**Figure 1:**
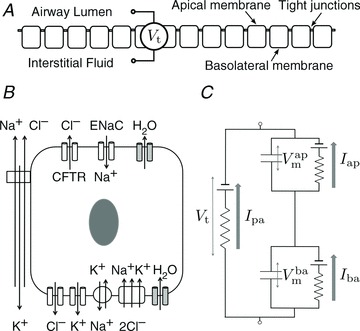
Schematic diagram of epithelial layer (*A*) and individual epithelial cell (*B*) separating the airway lumen from the interstitial fluid *A* and *B*, electrolyte transport occurs across the apical and basolateral membranes, and along the paracellular path through tight junctions. Transport parameters characterise flux through each pathway: CFTR and ENaC channels in the apical membrane are characterised by apical Cl^−^ permeability (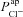) and apical Na^+^ permeability(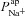), respectively; K^+^ and Cl^−^ channels in the basolateral membrane are characterised by the basolateral K^+^ (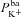) and Cl^−^ (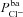) permeabilities; the transport parameters for the Na^+^–K^+^-ATPase pump proteins and NKCC cotransport proteins in the basolateral membrane are their densities per unit area of the membrane, ρ_NaK_ and ρ_NKCC_, respectively. The state of the cell at any time is described by six variables, cell volume (*W*_i_), moles of Na^+^, Cl^−^ and K^+^ in the cell (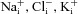, respectively), and apical (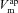) and basolateral (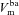) membrane potentials. *C*, equivalent electrical circuit representation of airway epithelium. 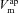 and 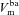 are coupled electrically via the current along the paracellular pathway (*I*_pa_). The trans-epithelial potential difference *V*_t_ is given by the difference between lumen and serosal potential (i.e. 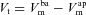).

Our mathematical model simulates a monolayer of HNE cells placed between two well-perfused compartments containing physiological saline solution (Fig. 1*A*), thus approximating the environment experienced by HNE cells *in vivo* during nasal *V*_t_ measurements when the airway surface is flooded (or *in vitro* during an Ussing chamber experiment). In this model Na^+^, Cl^−^, K^+^ and water move between interstitial fluid and airway lumen (paracellular route) and between the cell and external solutions via transport processes in the apical and basolateral plasma membranes (Fig. 1*B*). The magnitude of the ion flux due to each of these component processes in the model is proportional to a *transport parameter* that is related to the density of that component in the plasma membrane.

We calculate the flux of ions from each individual transport pathway as a function of the driving force and associated transport parameter, and employ an equivalent electrical circuit description of the epithelium to determine membrane (apical, 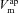 and basal, 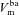) and trans-epithelial (*V*_t_) potentials in the open circuit configuration (Fig. 1*C*). This framework allows us to vary transport parameters or extracellular solution composition, and calculate the resultant changes in membrane potentials and *V*_t_ in a quantitative manner. It also allows a quantitative investigation of how these responses to perturbations change when transport processes are varied.

### Transport pathways included in model

There are four ion channel components included in the model. ENaC and CFTR channels will give rise to apical Na^+^ (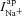) and Cl^−^ (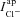) currents, respectively, and basolateral K^+^ and Cl^−^ channels facilitate the basolateral currents 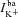 and 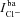. Apical K^+^ channels are not included since they do not contribute substantially to *V*_t_ (Knowles *et al.* 1983; Willumsen *et al.* 1989*a*). Channel currents were modelled using the Goldman–Hodgkin–Katz (GHK) flux equation (Hille, 2001), which relates the trans-membrane electrochemical driving force (determined by the membrane potential and concentration gradient) to the trans-membrane current, given the permeability of the membrane to a particular ion (see Supplemental material, section S1, available online only). For example, given apical membrane potential (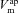), lumen and intracellular Na^+^ concentrations ([Na^+^]_l_ and [Na^+^]_i_), and the permeability of the apical membrane to Na^+^ (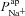), we can compute the ENaC current (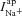). Paracellular ion currents (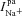, 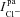, 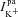, 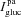) are also modelled using the GHK equation (with *V*_t_ as electrical driving force, and ion concentrations from the luminal and serosal compartments).

We include descriptions of the Na^+^-K^+^–2Cl^−^ co-transport protein NKCC1 and Na^+^–K^+^-ATPase pump protein in our model, which generate the basolateral ion fluxes *J*_NKCC_ and *J*_NaK_, respectively. We use the model of Benjamin & Johnson to calculate flux from the Na^+^–K^+^–2Cl^−^ co-transporter (Benjamin & Johnson, 1997), and the model of Smith & Crampin to describe active transport by the Na^+^–K^+^-ATPase (Smith & Crampin, 2004; see Supplemental material, section S1, for full details). The total flux along these transport pathways is proportional to the density of the relevant protein in the basolateral membrane, ρ_NKCC_ and ρ_NaK_.

In our model both apical and basolateral membranes are permeable to water. The trans-membrane water flux in both cases (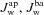) is assumed to be proportional to the trans-membrane osmolarity gradient Δ*S*, where the osmolarity here is given by the total Na^+^, Cl^−^ and K^+^ concentrations as well as the concentration of impermeable anions in that given compartment.

### Transport kinetics

Cellular variables evolve in time based on the net influx or efflux of ions and water, and we described these kinetics with a system of coupled, non-linear ordinary differential equations.

Cell volume *W*_i_ changes if there is a net influx or efflux of water (water flux is positive in serosal to mucosal direction):


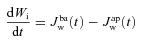
(1)

The ionic composition of the intracellular compartment changes due to the net trans-membrane ion fluxes (positive ion currents denote a flux of positive ions *out* of the cell, positive *J*_NKCC_ denotes ion flux *into* the cell, *F* is the Faraday constant and *z_n_* is the valence of the ion *n* under consideration):


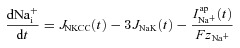
(2)


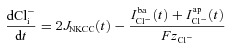
(3)


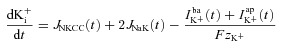
(4)

The equivalent electrical circuit description of the epithelium (Fig. 1*C*) can be used to calculate how the membrane potentials change due to net apical, basolateral and paracellular currents (*C*_m_ is the capacitance per unit area of the plasma membrane):


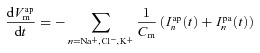
(5)


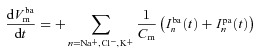
(6)

The trans-epithelial potential difference, with the serosal compartment as the earth, is given by 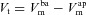 (see Supplemental material, section S1).

### Baseline transport parameter values

We initially found estimates of transport parameters (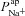, 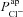, 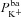, ρ_NaK_, ρ_NKCC_, 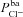) from the relevant scientific literature, and refer to these as baseline parameter values (Table 1; note that here we assume that the paracellular permeability, *P*_pa_, is non-selective and does not change in CF; the rationale for this is discussed later; see also Supplemental material, section S4). These were used to give an order of magnitude estimate for each parameter and thus initially identify the region of parameter space on which our parameter estimation should focus.

**Table 1:** Baseline values used (column 3) and numerical estimates found for parameter values in non-CF and CF cells (columns 5 and 6) for each free transport parameter (rows 1–6)

Parameter	Units	Baseline	Reference	Non-CF	CF
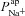	μm s^−1^	0.028	(Willumsen & Boucher, 1991*b*)	0.024	0.065
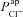	μm s^−1^	0.072	(Willumsen & Boucher, 1991*b*)	0.066	0.006
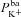	μm s^−1^	0.080	(Falkenberg & Jakobsson, 2010)	0.103	0.400
ρ_NaK_	10^−10^ mol cm^−2^	0.400	*	0.127	0.489
ρ_NKCC_	10^−10^ mol cm^−2^	0.400	*	0.188	2.000
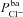	μm s^−1^	0.100	(Falkenberg & Jakobsson, 2010)	0.097	0.144

* ρ_NaK_ and ρ_NKCC_ estimated by authors.

## Results

### CF epithelia have an increased 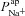


We set out to determine, then to compare, the value of 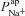 in CF and non-CF nasal epithelial cells. To constrain the model we used extensive data sets obtained from cultured HNE cells, including time course data covering the addition of amiloride or reduction of [ Cl^−^]_l_ at the apical membrane (Willumsen *et al.* 1989*a*,*b*; Willumsen & Boucher, 1991*a*,*b*). We thus formulated an optimisation problem to minimise the residual errors between physiological properties predicted by the mathematical model, and those observed experimentally, by varying transport parameters of interest (see Supplemental material, section S2, for details).

**Figure 2:**
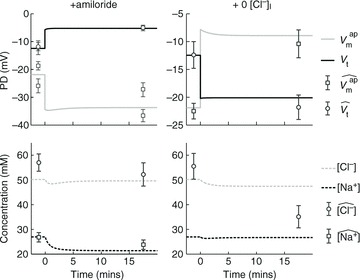
Estimating transport parameters from electrophysiological recordings Model predictions for 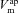, *V*_t_, [ Na^+^]_i_ and [ Cl^−^]_i_ (continuous and dashed lines) are plotted for simulations of ‘+amiloride’ and ‘+ 0[ Cl^−^]_l_’ experiments, and compared with their observed values (symbols). Data used are from non-CF HNE cells, and parameter values used for the simulation are those which were found to minimise the residual error between model output and these data (Table 1).

Using simulations made with parameter values optimised for non-CF epithelia, our model accurately fits the observed initial and final steady-state values for membrane and trans-epithelial potentials (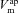, 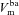 and *V*_t_) and concentrations ([ Na^+^]_i_, [ Cl^−^]_i_) both in amiloride addition and low Cl ^−^ experiments (Fig. 2). A similar analysis was carried out to identify parameter values best describing corresponding experimental data obtained on CF epithelia (Supplemental Fig. S3). The optimised parameter values for non-CF and CF epithelia are shown in Table 1.

The optimal parameter values obtained for ENaC and CFTR permeability are similar to those estimated experimentally (Table 1). Examining the difference between optimal CF and non-CF parameter values, we found not only that in CF 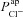 must be reduced (as expected) but also that the value of 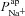 must be significantly increased.

Very little experimental data are available on the magnitude and characteristics of paracellular permeability. In order to determine if increased 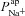 in CF epithelia was dependent on assumptions we had made regarding paracellular ion transport, we repeated the parameter estimation analysis assuming a lower *P*_pa_ in CF (Willumsen & Boucher, 1989) and/or a cation-selective paracellular transport (Levin *et al.* 2006; Flynn *et al.* 2009). We found that while these differences in paracellular transport do have an influence over the exact value of 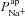 or 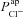 estimated, they do not alter how each of these parameters changes in CF relative to non-CF epithelia (see Supplemental Tables S5 and S6).

### Feasible ranges of 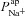 differ between populations of non-CF and CF nasal epithelial cells

Although our optimisation results provide good evidence for a change in 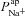, we were conscious that the data which we used for fitting in the optimisation problem were the mean of several experiments carried out on different primary cultures of HNE cells. Variations in the experimental results obtained from these cells show that a large range of values of, for example, intracellular [ Na^+^], are physiologically reasonable. We wanted to make sure that by optimising parameter fits to average data we did not exclude parameter sets that could account for both CF and non-CF data given the full range of possible variation (e.g. Willumsen & Boucher, 1991*b*).

**Figure 3:**
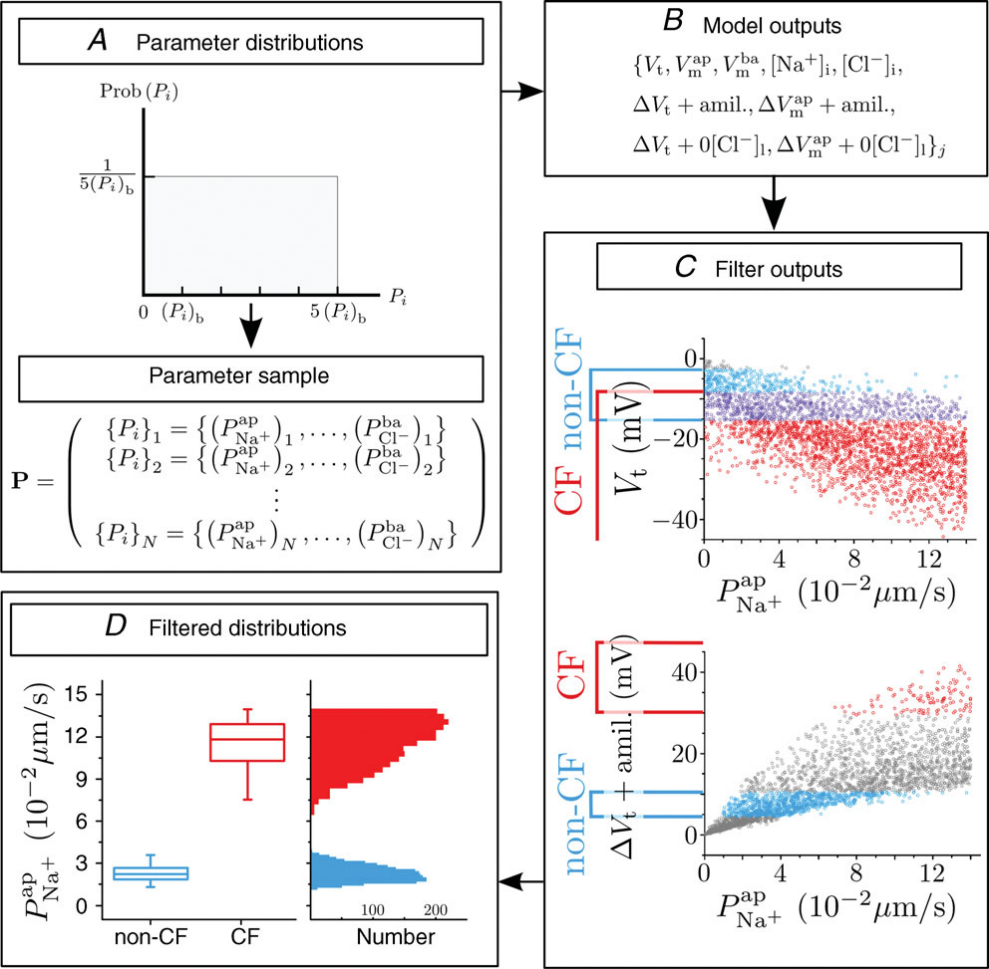
Monte Carlo filtering analysis approach to determine distributions of transport parameter values in CF and non-CF HNE cells *A*, a large sample (*N*= 10^6^) of parameter sets was generated, each set is a vector 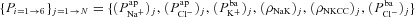. Each parameter *i* is sampled from a uniform distribution *U*(0, 5(*P_i_*)_b_) around its baseline value (see Table 1). *B*, for each parameter set {*P_i_*}_*j*_, the values of variables *X_j_* at steady state and their value after ‘+amiloride’ and ‘+ 0[ Cl^−^]_l_’ perturbations are calculated. *C*, bounds are placed on the allowed values of these model outputs, for both CF and non-CF states, and parameter sets are classified on this basis. *D*, filtered parameter distributions can be examined to assess how transport parameters vary between normal and disease states.

To achieve this, we carried out a large number of simulations with the model, as illustrated schematically in Fig. 3. We first used Monte Carlo sampling to randomly generate 10^6^ parameter sets, sampling values for each transport parameter (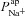, 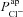, 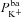, ρ_NaK_, ρ_NKCC_, 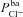) from a uniform distribution on a bounded region (from zero to five times) around the relevant baseline parameter value (Fig. 3*A*). This process provided a population of model parameter sets, each with a unique set of parameter values, steady-state variable values, kinetic properties and so on (Fig. 3*B*). We next separated the sample population (Fig. 3*C*) into 1975 parameter sets which predicted observed steady-state and kinetic properties of non-CF HNE cells and 2430 which reproduced the observed steady-state and kinetic behaviour of CF HNE cells (see Table 2 for both non-CF and CF filtering bounds). The other parameter sets which produced non-physiological values or unstable kinetics were discarded (see Supplemental material, section S3, for full details).

**Table 2:** Constraints on allowed variable values in CF and non-CF cells, based on data from primary cultures of HNE cells

		Non-CF			CF		
					
Property	Units	Lower	Upper	Source	Lower	Upper	Source
[Na^+^]_i_	mm	18.0	43.2	(Willumsen & Boucher, 1991*b*)	21.0	51.3	(Willumsen & Boucher, 1991*a*)
[Cl^−^]_i_	mm	32.5	84.4	(Willumsen *et al.* 1989*a*)	32.5	84.4	(Willumsen *et al.* 1989*b*)
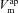	mV	−38.6	−14.9	(Willumsen & Boucher, 1991*b*)	−37.7	6.7	(Willumsen & Boucher, 1991*a*)
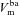	mV	−45.1	−24.2	(Willumsen & Boucher, 1991*b*)	−59.3	−33.6	(Willumsen & Boucher, 1991*a*)
*V*_t_	mV	−15.5	−2.7	(Willumsen & Boucher, 1991*b*)	−59.2	−8.2	(Willumsen & Boucher, 1991*a*)
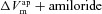	mV	−14.0	−5.5	(Willumsen *et al.* 1989*a*; Willumsen & Boucher, 1991*b*)	−47.4	−29.0	(Willumsen *et al.* 1989*b*; Willumsen & Boucher, 1991*a*)
Δ*V*_t_+ amiloride	mV	4.7	10.1	(Willumsen *et al.* 1989*a*; Willumsen & Boucher, 1991*b*)	30.1	47.1	(Willumsen *et al.* 1989*b*; Willumsen & Boucher, 1991*a*)
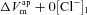	mV	9.2	15.0	(Willumsen *et al.* 1989*a*)	−5.3	11.1	(Willumsen *et al.* 1989*b*)
Δ*V*_t_+ 0[ Cl^−^]_l_	mV	−12.7	−6.1	(Willumsen *et al.* 1989*a*)	−16.5	9.9	(Willumsen *et al.* 1989*b*)

**Figure 4:**
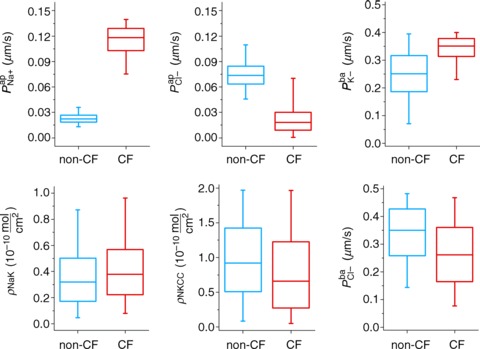
Distributions of each transport parameter, found by constraining allowed model behaviour in non-CF (blue) and CF (red) states In sequence from low to high, features denoted in each boxplot are: 1st, 25th, 50th (median), 75th and 99th percentiles of the given parameter's distribution.

Figure 4 illustrates the distributions of transport parameter values which remain after applying the non-CF (blue) and CF (red) filters (see also Supplemental material Figs S4 and S5, and Tables S5 and S6, respectively). While the non-CF and CF distributions are similar for some parameters, the distributions of 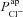 and 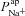 differ markedly, CFTR permeability being decreased and ENaC permeability increased in the disease state. Thus, extending our analysis to take into account the full distribution of allowed cellular variable values, rather than focusing on mean behaviour, confirms that ENaC permeability must be increased in CF relative to non-CF cells, in order to explain the observed quantitative differences in electrophysiological properties.

We repeated this Monte Carlo filtering analysis to determine whether or not decreased paracellular permeability in CF, and/or selectivity of the paracellular pathway, would significantly alter these conclusions. Again we found this was not the case: neither a higher shunt resistance in CF nor a cation-selective paracellular pathway affected our conclusions that permeability distributions were shifted in CF epithelia, with median CFTR permeability decreased and median ENaC permeability increased (see Supplemental material, section S4, Figs S6–S8).

### Hyperpolarised *V*_t_ in CF can be explained by increased 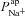, but not by reduced 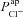


**Figure 5:**
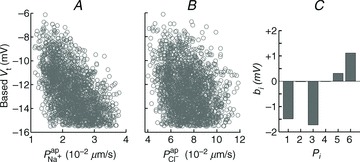
Sensitivity analysis investigating the influence of model parameters on *V*_t_ A multiple regression of the form 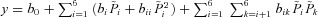 was fitted using the set of normalised parameter values 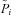 in the non-CF parameter distribution as regressors, and the corresponding model output *y*={*V*_t_} as the independent variable (see Supplemental material, section S4). Basal *V*_t_ is plotted as a function of 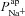 (*A*) and 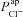 (*B*), for the 1975 parameter sets belonging to the non-CF distribution. *C*, strength of linear interaction (*b_i_*) between transport parameters 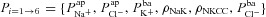 and *V*_t_, found via sensitivity analysis. 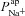 hyperpolarises *V*_t_ (*b*_1_=−1.49 mV), but changing 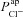 has little influence (*b*_2_=−0.02 mV).

**Figure 6:**
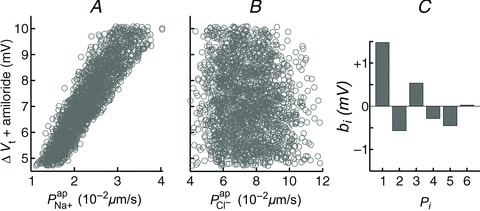
Sensitivity analysis investigating the influence of model parameters on Δ*V*_t_+ amiloride Amiloride-sensitive *V*_t_ is plotted against 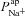 (*A*) and 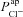 (*B*), for their non-CF distributions. *C*, sensitivity analysis results plotting strength of interaction (*b_i_*) between Δ*V*_t_+ amiloride and transport parameters 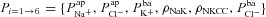. 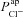 tends to decrease this metric, despite its limited effect on basal *V*_t_.

To further investigate the functional relationship between each individual transport parameter, *P_i_*, and the epithelial bioelectric properties in question (i.e. model output variables basal *V*_t_, Δ*V*_t_+ amiloride, and Δ*V*_t_+ 0[ Cl^−^]_l_) we carried out a variance-based sensitivity analysis (Sobie, 2009; Taylor *et al.* 2009) using the 1975 parameter sets in the non-CF distribution along with their model outputs (see Figs 5 and 6, and Supplemental material, section S4). The coefficients we obtained (Figs 5*C* and 6*C*) gave us an objective means of quantifying the relative influence of each transport parameter on these bioelectric properties.

Figure 5*A* and *B* shows scatter plots of 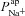 and 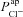, respectively (from the non-CF parameter value distributions), against basal (steady state) *V*_t_ predicted by each. Figure 5*C* summarises the results of the sensitivity analysis. There is a negative correlation between 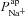 and *V*_t_ apparent in panel *A*, and confirmed by the large negative regression coefficient *b*_1_ (−1.49 mV) in panel *C*. A significant correlation between 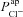 and *V*_t_ is not clear in panel *B*. The sensitivity analysis confirms that while 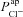 does influence *V*_t_ to an extent, on average over this region of parameter space it has a much smaller effect than 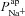, 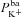 or 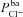 (regression coefficient *b*_2_=−0.02 mV, <2%*b*_1_). Therefore an increase in 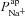 is necessary to hyperpolarise basal *V*_t_ to the values seen in CF epithelia, while changes in 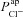 do not influence *V*_t_ to the same extent.

### Amiloride-sensitive *V*_t_ is inversely related to 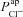, but is more strongly influenced by 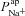


Figure 6*A* and *B* illustrates the relationship between the 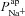 and 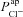 parameter values, respectively, from the non-CF distributions, and the corresponding predicted Δ*V*_t_+ amiloride. Not surprisingly, there is a positive correlation between 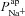 and Δ*V*_t_+ amiloride. However, the relationship between Δ*V*_t_+ amiloride and 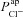 is less obvious. The results of the sensitivity analysis in Fig. 6*C* show that 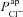 has the second greatest influence on Δ*V*_t_+ amiloride. While increasing 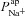 tends to increase the magnitude of Δ*V*_t_+ amiloride, increasing 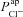 tends to decrease its magnitude.

### Loss of Cl^−^ conductance can hyperpolarise basal *V*_t_, but not to the extent seen in CF

It is clear that 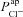 can influence basal *V*_t_, even if it does not do so to the same extent as 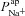. We wanted to determine, quantitatively, what magnitude of a change in basal *V*_t_ the model would predict upon loss of 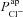 alone, and compare this to the hyperpolarisation of *V*_t_ observed in CF. Therefore, for each parameter set producing plausible physiological values in the non-CF distribution, we set 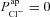 and found the new steady state of the system. We define 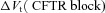 as the difference between this new *V*_t_, and the initial basal *V*_t_ when 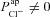.

We can analyse the magnitude of 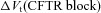 and its relationship to Δ*V*_t_+ 0[ Cl^−^]_l_ (change in *V*_t_ induced by reducing [ Cl^−^]_l_) which is commonly used as a measure of the underlying Cl^−^ conductance (see Supplemental Fig. S9). For a given Δ*V*_t_+ 0[ Cl^−^]_l_, CFTR loss can depolarise or hyperpolarise *V*_t_, depending on the magnitudes of the other transport parameter values. The average Δ*V*_t_+ 0[ Cl^−^]_l_ observed experimentally in non-CF HNE cells was not greater than –15mV (Willumsen *et al.* 1989*a*), a value close to that reported *in vivo*, in nasal PD measurements. However, the maximum hyperpolarisation achieved by blocking 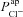 was around −4 mV. This is much smaller in magnitude than the average hyperpolarisation seen in CF patients (and in primary cultures of CF HNE cells; Willumsen & Boucher, 1991*b*), which is around −20 mV (Knowles *et al.* 1995).

## Discussion

We have developed a mathematical model of ion transport in human nasal epithelial cells. As the nasal epithelium is the site of *in vivo* measurements made on patients, it has been well characterised (Willumsen & Boucher, 1989, 1991*b*; Willumsen *et al.* 1989*b*) and is clinically important in the assessment of CF (Simmonds *et al.* 2011). The advantages of specifically modelling nasal epithelia are thus 2-fold. First, it offers the opportunity to exploit a very large body of existing measurements for validation and parameter estimation purposes. Second, by leading to a better quantitative analysis of nasal potential difference measurements, it improves our understanding of CF disease.

The agreement between our model predictions and the known physiology, in terms of capturing essential changes in membrane potentials and intracellular ion concentrations (Fig. 2), suggests that the model provides a realistic picture of the major epithelial ion transport processes which determine nasal trans-epithelial potential. Fitting the model to experimental data, we observed not only that 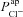 must be reduced, but also that 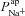 had to increase (Table 1) to account for the bioelectric properties of CF epithelial cells. This prediction also held when we ran multiple simulations to take into account cell variability: parameter sets resulting in steady-state and kinetic characteristics typical of CF cells included not only reduced 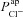 but also increased 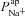 (Fig. 4). The fact that our analysis did not make any initial judgements regarding how parameters should vary in the disease state, and hence did not bias us towards computing these results, gives us confidence in their validity and further demonstrates that the findings regarding ENaC and CFTR permeabilities are robust.

### Impact of model assumptions

Inevitably, when describing a complex biological system with a mathematical model, one makes a number of assumptions in order to concentrate on the phenomena of interest, and to keep the analysis tractable. The main assumption we make is that Na^+^, Cl^−^ and K^+^ currents largely determine nasal *V*_t_ and that the membrane permeabilities of these ions can be estimated from trans-epithelial electrical recordings. In making this simplification we implicitly assume that bicarbonate transport does not substantially impact *V*_t_. In our analysis we saw that 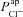 has little effect on basal *V*_t_ (Fig. 5), and limited effect on Δ*V*_t_+ amiloride (Fig. 6*B* and *C*). It is therefore likely that including bicarbonate transport would not alter this picture, as there is significantly less HCO_3_^−^ transport through CFTR channels than Cl^−^ (Poulsen *et al.* 1994). Indeed a very recent paper carries out a similar analysis for non-CF nasal epithelia and essentially validates this approach (Garcia *et al.* 2013).

Initially, we also made the assumption that changes in paracellular permeability do not drive the bioelectric changes observed in CF. Later, by relaxing this condition, we found that while differences in paracellular permeability or selectivity do influence estimates of 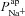 and 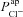, they do not alter how each of these parameters changes in CF relative to non-CF epithelia (see Supplemental material Tables S5 and S6, Figs S6–S8). The magnitude of the increase in 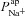 will therefore be influenced by an increased shunt resistance (2-fold rather than 3-fold increase), but the increase of this transport parameter in the disease state is observed consistently.

### Quantifying the influence of CFTR and ENaC currents on nasal *V*_t_

Published modelling work investigating the electrical coupling of CFTR and ENaC fluxes (Horisberger, 2003) showed that increasing 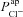 could decrease amiloride-sensitive short-circuit current (*I*_sc_) in a kidney epithelial cell model, and Falkenberg and Jakobsson note that *I*_sc_ is most sensitive to basal apical anion permeability, after the addition of amiloride (Falkenberg & Jakobsson, 2010). More recently, evidence from pig and human airway epithelial cell lines showed that experimentally decreasing apical Cl^−^ conductance can increase Δ*V*_t_+ amiloride (Chen *et al.* 2010; Itani *et al.* 2011). Our analyis confirms that this relationship exists, qualitatively. However, our modelling approach allows us to quantitatively determine the influence each transport parameter has on the electrical properties of the epithelium (Figs 5 and 6). Thus, we can show that the magnitude of changes in going from non-CF to CF levels of anion permeability were not sufficient to explain the experimentally observed hyperpolarised basal *V*_t_, the increased amiloride-sensitive *V*_t_ component, and the decreased Δ*V*_t_+ 0[ Cl^−^]_l_. In contrast, sensitivity analysis shows that 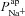 significantly hyperpolarises basal *V*_t_, and is the most important factor in determining the magnitude of Δ*V*_t_+ amiloride. Without altering 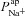 from non-CF levels, the magnitude of the hyperpolarisation of basal *V*_t_ and of increased amiloride-sensitive *V*_t_ could not be explained.

One can intuitively understand how the relative influences of ENaC and CFTR permeability on basal and amiloride-sensitive *V*_t_ arise, by examining the driving force for movement of Na^+^ and Cl^−^ ions across the apical membrane. Basal *V*_t_ depends implicitly on apical Na^+^ and Cl^−^ currents, and the changes in these currents with respect to permeability are proportional to driving force. Hence the relative driving force for movement of different ions explains the relative sensitivity of *V*_t_ to different permeabilities.

In the representative example of best-fit non-CF parameter values (Table 1), the driving force for Na^+^ absorption across the apical membrane at steady state is −65.8 mV, as opposed to +1.1 mV for Cl^−^ transport. At these physiological potentials, the Cl^−^ driving force is thus < 2% of that for Na^+^, consistent with the results of our sensitivity analysis: 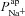 has a much greater influence on *V*_t_ than 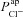. How then, can we explain the influence of 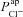 on amiloride-sensitive *V*_t_? After amiloride is added 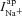 is dramatically reduced and 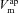 changes, altering the apical Cl^−^ driving force and consquently 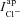. Again taking these best-fit parameters, this driving force goes from +1.1 mV to −9.5 mV for Cl^−^, while 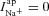. Therefore 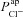 now has a greater relative influence on 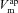 and *V*_t_, while 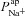 can have no further effect.

The results of our sensitivity analysis are in agreement with a range of additional experimental data not used to constrain the model. For example, we observed basal *V*_t_ to be strongly dependent on 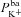 (hyperpolarising). This was found experimentally by Mall *et al.* (2000) who blocked basolateral K^+^ channels in human bronchial epithelial (HBE) cells. Modelling studies have also shown that *I*_sc_ can be increased by stimulating basolateral K^+^ currents (Falkenberg & Jakobsson, 2010), supporting the hypothesis that increased basolateral K^+^ conductance is necessary to hyperpolarise the basolateral (and consequently, apical) membrane, providing an increased driving force for Cl^−^ secretion (Cotton, 2000). Further, *V*_t_ tends to be depolarised by 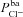 in our model, which agrees with the observations of Fischer and colleagues in human and bovine tracheal primary cultures (Fischer *et al.* 2007) who also found *V*_t_ to be dependent on 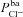.

Finally, it is interesting to note how our model predicts that the density of Na^+^–K^+^-ATPase pumps is higher in CF than non-CF cells. This may be necessary in order to deal with the increased rate of Na^+^ absorption, and higher pump expression has been reported in CF tracheal and nasal epithelia (Stutts *et al.* 1986).

### Implications for clinical nasal potential difference measurements

**Figure 7:**
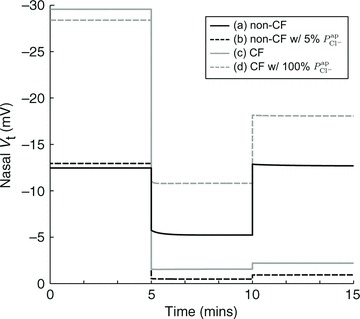
Simulations of first stages of a clinical nasal PD test Basal *V*_t_ is recorded initially for several minutes, then amiloride is added to the perfusing solution at *t* = 5 min to block ENaC channels (causing 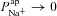), and the resultant change in *V*_t_ is recorded. At *t* = 10 min the solution perfusing the luminal surface is changed to a low Cl^−^ solution ([ Cl^−^]_l_→ 3 mm) to introduce a diffusion potential for Cl^−^ efflux, and the resultant change in *V*_t_ is recorded. Restoring non-CF 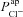 levels in a CF cell does not correct hyperpolarised basal *V*_t_ (grey continuous line → grey dashed line). Also, reducing 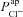 alone to CF levels, in a non-CF cell, does not reproduce a typical CF trace (black continuous line → black dashed line).

In Fig. 7 we show the output of simulations of the first three stages of a standard nasal potential difference (nasal *V*_t_) clinical recording: measurement of a basal *V*_t_ value, relaxation to a new steady-state value following apical amiloride addition, and transition to a third *V*_t_ value upon transfer to Cl^−^-free conditions (while maintaining amiloride presence). Simulations were run with four different parameterisations: (a) optimal non-CF values (black), (b) optimal non-CF with reduced 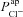 (5% of optimal level, black, dashed), (c) optimal CF values (grey), and (d) optimal CF values with optimal non-CF 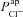 levels (grey, dashed). These simulations illustrate the major findings of our study, and emphasise the potential utility of this mathematical modelling approach.

Traces (a) and (b) illustrate how the loss of apical Cl^−^ permeability (in a non-CF HNE cell) alone cannot account for CF bioelectric properties. With a reduction to 5% of non-CF 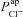 levels, the level of Δ*V*_t_+ amiloride increases and Δ*V*_t_+ 0[ Cl^−^]_l_ decreases, but the change of tens of millivolts in basal *V*_t_ seen in patients is not observed as only a modest hyperpolarisation occurs.

Traces (c) and (d) illustrate how our model can be used to investigate strategies aimed at normalising ion transport in CF epithelia. Here we simulate the effect of theoretically increasing 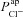 from CF to non-CF levels, in a CF HNE cell. We see that this can ameliorate the Δ*V*_t_+ amiloride and Δ*V*_t_+ 0[ Cl^−^]_l_ responses towards non-CF magnitudes, but basal *V*_t_ remains hyperpolarised at a typical CF level. This simulation investigates the changes in *V*_t_ caused by a hypothetical therapy aimed at increasing Cl^−^ secretion alone. Although the exact pathophysiology of CF lung disease is controversial, the hyperpolarised *V*_t_ experienced by CF epithelia will undoubtedly alter driving forces for trans-epithelial ion (and water) movement, a factor which may contribute to the development of CF lung disease. Thus we can see that such a strategy (e.g. stimulating calcium-activated Cl^−^ channels in the apical membrane; Cuthbert, 2011) would not help with restoring basal *V*_t_ in native tissues to desired non-CF levels.

The measurement of nasal trans-epithelial potentials is widely used as an aid to CF diagnosis and clinical management. Hyperpolarised basal *V*_t_ and larger amiloride-sensitive *V*_t_ changes are hallmarks of CF disease and have, in recent years, also become central to the debate on the role of sodium hyper-absorption in CF pathology. These same altered bioelectric properties are the foundation of therapeutic approaches aimed at reducing ENaC activity (Hofmann *et al.* 1998; Coote *et al.* 2009). The correct interpretation of trans-epithelial potentials therefore carries important implications for both understanding CF and assessing potential therapies. The model presented here therefore can become a highly valuable tool for the interpretation of clinical nasal potential difference measurements and for the development of more effective treatment.

## Abbreviations

CF: cystic fibrosis
CFTR: cystic fibrosis transmembrane conductance regulator
ENaC: epithelial Na^+^ channel
PD: potential difference
*V*_t_: trans-epithelial PD
HNE cells: human nasal epithelial cells
