# Supplementary material for: Increased apical Na+ permeability in cystic fibrosis is supported by a quantitative model of epithelial ion transport
Source: J Physiol. 2013 Jun 3;591(Pt 15):3681–92. doi: 10.1113/jphysiol.2013.253955 (PMC3752450; doi:10.1113/jphysiol.2013.253955)
Supplement: Supplementary file 1 [file tjp0591-3681-SD1.pdf]

Supplementary Material

Increased apical Na^+ permeability in cystic fibrosis is supported by a quantitative model of epithelial ion transport

O'Donoghue, Donal L [1], Dua, Vivek [2], Moss, Guy W J [1,3] Vergani, Paola [3,*]

(1) Centre for Mathematics and Physics in the Life Sciences and Experimental Biology, University College London, Gower Street, London, WC1E 6BT, United Kingdom.

(2) Department of Chemical Engineering, University College London, Gower Street, London, WC1E 6BT, United Kingdom.

(3) Department of Neuroscience, Physiology & Pharmacology, University College London, Gower Street, London, WC1E 6BT, United Kingdom.

Contents

S1 Equations and fixed parameters in mathematical model 2

Equation list 2

GHK flux equation 3

NKCC co-transporter model 4

Na^+-K^+ pump model 5

Determining paracellular permeability from shunt resistance 7

S2 Model validation 10

S3 Numerical parameter estimation 12

S4 Determining feasible non-CF and CF parameter distributions 15

Monte Carlo sampling of parameter values from baseline estimates 15

Physiologically realistic steady state behaviour 15

Physiologically realistic transport kinetics 16

Influence of paracellular permeability & selectivity on feasible parameter distributions 18

S5 Model sensitivity analysis 19

S6 References 21

S7 Tables 23

S8 Figures 27

S1 Equations and fixed parameters in mathematical model

Equation list

A full list of the notation used in equations S1-S18 is given in Table S1, and the value of constants and parameters which are fixed in the model are shown in Table S2.

$$I_{Na^+}^{ap}(t) = P_{Na^+}^{ap} V_m^{ap}(t) \frac{F^2}{RT} \left(\frac{\gamma\, Na_i^+(t)/W(t) - \gamma [Na^+]_l \exp\left(-z_{Na^+} F \frac{V_m^{ap}(t)}{RT}\right)}{1 - \exp\left(-z_{Na^+} F \frac{V_m^{ap}(t)}{RT}\right)} \right) \quad \text{(S1)}$$

$$I_{Cl^-}^{ap}(t) = P_{Cl^-}^{ap} V_m^{ap}(t) \frac{F^2}{RT} \left(\frac{\gamma\, Cl_i^-(t)/W(t) - \gamma [Cl^-]_l\, exp\left(-z_{Cl^-} F \frac{V_m^{ap}(t)}{RT}\right)}{1 - exp\left(-z_{Cl^-} F \frac{V_m^{ap}(t)}{RT}\right)} \right) \quad \text{(S2)}$$

$$I_{K^+}^{ba}(t) = P_{K^+}^{ba} V_m^{ba}(t) \frac{F^2}{RT} \left(\frac{\gamma\, K_i^+(t)/W(t) - \gamma [K^+]_s \exp\left(-z_{K^+} F \frac{V_m^{ba}(t)}{RT}\right)}{1 - \exp\left(-z_{K^+} F \frac{V_m^{ba}(t)}{RT}\right)} \right) \quad \text{(S3)}$$

$$I_{Cl^-}^{ba}(t) = P_{Cl^-}^{ba} V_m^{ba}(t) \frac{F^2}{RT} \left(\frac{\gamma\, Cl_i^-(t)/W(t) - \gamma [Cl^-]_s \exp\left(-z_{Cl^-} F \frac{V_m^{ba}(t)}{RT}\right)}{1 - \exp\left(-z_{Cl^-} F \frac{V_m^{ba}(t)}{RT}\right)} \right) \quad \text{(S4)}$$

$$I_{Na^+}^{pa}(t) = P_{pa}(-V_t(t)) \frac{F^2}{RT} \left(\frac{\gamma [Na^+]_l - \gamma [Na^+]_s \exp\left(-z_{Na^+} F \frac{(-V_t(t))}{RT}\right)}{1 - \exp\left(-z_{Na^+} F \frac{(-V_t(t))}{RT}\right)} \right) \quad \text{(S5)}$$

$$I_{K^+}^{pa}(t) = P_{pa}(-V_t(t)) \frac{F^2}{RT} \left(\frac{\gamma [K^+]_l - \gamma [K^+]_s \exp\left(-z_{K^+} F \frac{(-V_t(t))}{RT}\right)}{1 - \exp\left(-z_{K^+} F \frac{(-V_t(t))}{RT}\right)} \right) \quad \text{(S6)}$$

$$I_{Cl^-}^{pa}(t) = P_{pa}(-V_t(t)) \frac{F^2}{RT} \left(\frac{\gamma [Cl^-]_l - \gamma [Cl^-]_s \exp\left(-z_{Cl^-} F \frac{(-V_t(t))}{RT}\right)}{1 - \exp\left(-z_{Cl^-} F \frac{(-V_t(t))}{RT}\right)} \right) \quad \text{(S7)}$$

$$I_{gluc.}^{pa}(t) = P_{pa}(-V_t(t)) \frac{F^2}{RT} \left(\frac{\gamma [gluc.]_l - \gamma [gluc.]_s \exp\left(-z_{gluc.} F \frac{(-V_t(t))}{RT}\right)}{1 - \exp\left(-z_{gluc.} F \frac{(-V_t(t))}{RT}\right)} \right) \quad \text{(S8)}$$

$$J_{NKCC}(t) = \rho_{NKCC} \frac{k_f^{full}\, k_f^{empty} \gamma^4 [Na^+]_e\, [K^+]_e\, [Cl^-]_e^2 - k_b^{full} k_b^{empty} \gamma^4\, [Na^+]_i\, [K^+]_i\, [Cl^-]_i^2}{\sum_{n=1}^{16} Z_{nkcc}^n} \quad \text{(S9)}$$

$$J_{NaK}(t) = \rho_{NaK} \frac{\alpha_1^+ \alpha_2^+ \alpha_3^+ \alpha_4^+ - \alpha_1^- \alpha_2^- \alpha_3^- \alpha_4^-}{\Sigma} \quad \text{(S10)}$$

$$J_w^{ap}(t) = L_w v_w \left([S]_l - \frac{Na_i^+(t) + Cl_i^+(t) + K_i^+(t) + \psi_i}{W(t)} \right) \quad \text{(S11)}$$

$$J_w^{ba}(t) = L_w v_w \left(\frac{Na_i^+(t) + Cl_i^+(t) + K_i^+(t) + \psi_i}{W(t)} - [S]_s \right) \quad \text{(S12)}$$

$$\frac{dW_i}{dt} = J_w^{ba}(t) - J_w^{ap}(t) \quad \text{(S13)}$$

$$\frac{dNa_i^+}{dt} = J_{NKCC}(t) - 3\, J_{NaK}(t) - I_{Na^+}^{ap}(t)/F\, z_{Na^+} \quad \text{(S14)}$$

$$\frac{dCl_i^-}{dt} = 2\, J_{NKCC}(t) - \left(I_{Cl^-}^{ba}(t) + I_{Cl^+}^{ap}(t) \right)/F\, z_{Cl^-} \quad \text{(S15)}$$

$$\frac{dK_i^+}{dt} = J_{NKCC}(t) + 2\, J_{NaK}(t) - \left(I_{K^+}^{ba}(t) + I_{K^+}^{ap}(t) \right)/F\, z_{K^+} \quad \text{(S16)}$$

$$\frac{dV_m^{ap}}{dt} = -\frac{1}{C_m} \left(I_{Na^+}^{ap}(t) + I_{Cl^-}^{ap}(t) + I_{Na^+}^{pa}(t) + I_{Cl^-}^{pa}(t) + I_{K^+}^{pa}(t) + I_{gluc}^{pa}(t) \right) \quad \text{(S17)}$$

$$\frac{dV_m^{ba}}{dt} = -\frac{1}{C_m} \left(-I_{K^+}^{ba}(t) - I_{Cl^-}^{ba}(t) - J_{NaK}(t) F z_{Na}^+ - I_{Na^+}^{pa}(t) - I_{Cl^-}^{pa}(t) - I_{K^+}^{pa}(t) - I_{gluc}^{pa}(t) \right) \quad \text{(S18)}$$

GHK flux equation

P_n^x is the permeability of membrane *x* per unit area to ion *n*, V_m^x is the electric PD across membrane *x*, *F* is the Faraday constant, *R* the universal gas constant, *T* is the temperature in Kelvin, and z_n is the valence of the ion in question. a_i^n is the thermodynamic activity of *n* in the cell (a_e^n is extracellular activity), which is related to its chemical concentration via $a_i^n = \gamma[n]_i$, where γ is the activity co-efficient. The current through ion channels in the apical and basolateral membranes can be determined quantitatively by substituting the appropriate values of V_m^x , P_n^x , $[n]_i$ and $[n]_e$ into equation S1-S4. Paracellular flux of Na^+ ($I_{Na^+}^{pa}$), K^+ ($I_{K^+}^{pa}$), Cl^- ($I_{Cl^-}^{pa}$) and gluconate (I_{gluc}^{pa}) can also be

modelled using the GHK formulism. For serosal to luminal paracellular current (flow of positive ions), the appropriate electrical driving force is $-V_t$, and the relevant concentrations are $[n]_l$ and $[n]_s$, which are used in equation S5-S8.

NKCC co-transporter model

Full description of kinetic model and parameter values used is given in (Benjamin & Johnson, 1997). We describe it briefly here.

The basolateral flux due to co-transport is given by

$$J_{NKCC}(t) = \rho_{NKCC}\, v_{NKCC}(t)$$

where ρ_{NKCC} (a free parameter in our model) is the number of co-transporters per unit area of the basolateral membrane, and $v_{NKCC}(t)$ is the turnover rate of a single co-transport protein, given by

$$v_{NKCC}(t) = \frac{k_f^{full}\, k_f^{empty} \gamma^4 [Na^+]_e\, [K^+]_e\, [Cl^-]_e^2 - k_b^{full} k_b^{empty} \gamma^4 [Na^+]_i\, [K^+]_i\, [Cl^-]_i^2}{\sum_{n=1}^{16} Z_{nkcc}^n}$$

The terms Z_{nkcc}^n are determined as follows

$Z_{nkcc}^1 = Z_1 \gamma [Cl^-]_i$

$Z_{nkcc}^2 = Z_2 \gamma [Na^+]_s$

$Z_{nkcc}^3 = Z_3 \gamma^2 [Cl^-]_i [K^+]_i$

$Z_{nkcc}^4 = Z_4 \gamma^2 [Cl^-]_s [K^+]_s$

$Z_{nkcc}^5 = Z_5 \gamma^3 [Cl^-]_i^2 [K^+]_i$

$Z_{nkcc}^6 = Z_6 \gamma^3 [Cl^-]_s [K^+]_s [Na^+]_s$

$Z_{nkcc}^7 = Z_7 \gamma^4 [Cl^-]_i^2 [K^+]_i [Na^+]_i$

$Z_{nkcc}^8 = Z_8 \gamma^4 [Cl^-]_s^2 [K^+]_s [Na^+]_s$

$Z_{nkcc}^9 = Z_9 \gamma^5 [Cl^-]_i^2 [K^+]_i [Na^+]_i\, [Na^+]_s$

$Z_{nkcc}^{10} = Z_{10} \gamma^5 [Cl^-]_i [Cl^-]_s^2 [K^+]_s [Na^+]_s$

$Z_{nkcc}^{11} = Z_{11} \gamma^6 [Cl^-]_i^2 [K^+]_i [Na^+]_i [Cl^-]_s [Na^+]_s$

$Z_{nkcc}^{12} = Z_{12} \gamma^6 [Cl^-]_i [K^+]_i [Cl^-]_s^2 [K^+]_s [Na^+]_s$

$Z_{nkcc}^{13} = Z_{13} \gamma^7 [Cl^-]_i^2 [K^+]_i [Cl^-]_i^2 [K^+]_s [Na^+]_s$

$Z_{nkcc}^{14} = Z_{14} \gamma^7 [Cl^-]_i^2 [K^+]_i [Na^+]_i [Cl^-]_s [K^+]_s [Na^+]_s$

$Z_{nkcc}^{15} = Z_{15}\gamma^8[Cl^-]_i^2[K^+]_i[Na^+]_i[Cl^-]_s^2[K^+]_s[Na^+]_s$

$Z_{nkcc}^{16} = Z_{16}\ K_{Cl}^2 K_K K_{Na}(k_b^{empty} + k_f^{empty})$

where the coefficients Z_n are given

$Z_1 = K_{Cl}K_K K_{Na}k_b^{empty}$

$Z_2 = K_{Cl}^2 K_k k_f^{empty}$

$Z_3 = K_{Cl}K_{Na}k_b^{empty}$

$Z_4 = K_{Cl}K_K k_f^{empty}$

$Z_5 = K_{Na}k_b^{empty}$

$Z_6 = K_{Cl}k_f^{empty}$

$Z_7 = k_b^{empty} + k_b^{full}$

$Z_8 = k_f^{full} + k_f^{empty}$

$Z_9 = K_{Na}^{-1}\ k_b^{full}$

$Z_{10} = K_{Cl}^{-1}\ k_f^{full}$

$Z_{11} = K_{Cl}^{-1}K_{Na}^{-1}k_b^{full}$

$Z_{12} = K_{Cl}^{-1}K_K^{-1}k_f^{full}$

$Z_{13} = K_{Cl}^{-2}\ K_K^{-1}\ k_b^{full}$

$Z_{14} = K_{Cl}^{-1}K_K^{-1}K_{Na}^{-1}k_b^{full}$

$Z_{15} = K_{Cl}^{-2}K_K^{-1}K_{Na}^{-1}(k_b^{full} + k_f^{full})$

$$k_b^{empty} = \frac{K_{Cl}^2\ K_K\ K_{Na}\ k_f^{full}\ k_f^{empty}}{K_{Cl}^2 K_K K_{Na} k_b^{full}}$$

Rate constants and dissociation constants used in this model are $K_{Cl} = 2.42$mM, $K_{Na} = 22.38$mM, $K_K = 234.74$mM, $k_f^{empty} = 37{,}767 s^{-1}$, $k_f^{full} = 1406\ \mathrm{s}^{-1}$, $k_b^{empty} = 13{,}196\ \mathrm{s}^{-1}$, $k_b^{full} = 4025\ \mathrm{s}^{-1}$, and they are also taken from reference (Benjamin & Johnson).

Na^+-K^+ pump model

Full description of sodium potassium pump model are available in reference (Smith & Crampin, 2004), we use their model description and parameter values. A brief description of the pump model is outlined here.

Flux from the sodium potassium pump, $J_{NaK}(t)$, is given by

$$J_{NaK}(t) = \rho_{NaK}\, v_{NaK}(t)$$

where $v_{NaK}(t)$ is the turnover rate of an individual pump protein, and ρ_{NaK} is the number of pump proteins per unit area of the basolateral membrane (free parameter in our model).

The turnover rate is given by

$$v_{NaK}(t) = \frac{\alpha_1^+\alpha_2^+\alpha_3^+\alpha_4^+ - \alpha_1^-\alpha_2^-\alpha_3^-\alpha_4^-}{\Sigma}$$

where $\alpha_{1,2,3,4}^{+/-}$ are the forward and backward rate constants of the reduced 4 stage pump cycle:

$$\alpha_1^+ = \frac{k_1^+\,\widetilde{Na_i^+}^3}{\left(1+\widetilde{Na_i^+}\right)^3 + \left(1+\widetilde{K_i^+}\right)^2 - 1}$$

$$\alpha_2^+ = k_2^+$$

$$\alpha_3^+ = \frac{k_3^+\,\widetilde{K_s^+}^2}{\left(1+\widetilde{Na_s^+}\right)^3 + \left(1+\widetilde{K_s^+}\right)^2 - 1}$$

$$\alpha_4^+ = \frac{k_4^+\widetilde{MgATP}}{1+\widetilde{MgATP}}$$

$$\alpha_1^- = k_1^-[MgADP]$$

$$\alpha_2^- = \frac{k_2^-\widetilde{Na_s^+}^3}{\left(1+\widetilde{Na_s^+}\right)^3 + \left(1+\widetilde{K_s^+}\right)^2 - 1}$$

$$\alpha_3^- = \frac{k_3^-[Pi][H^+]}{1+\widetilde{MgATP}}$$

$$\alpha_4^- = \frac{k_4^-\widetilde{K_i^+}^2}{\left(1+\widetilde{Na_i^+}\right)^3 + \left(1+\widetilde{K_i^+}\right)^2 - 1}$$

Normalised concentrations:

$$\widetilde{Na_i^+} = \gamma[Na^+]_i/k_{d,Na_i^+}$$

$$\widetilde{K_i^+} = \gamma[K^+]_i/k_{d,\,K_i^+}$$

$$\widetilde{Na_s^+} = \gamma[Na^+]_s/k_{d,Na_s^+}$$

$$\widetilde{K_s^+} = \gamma[K^+]_s/k_{d,K_s^+}$$

$$\widetilde{MgATP} = [MgATP]/k_{d,MgATP}$$

Voltage dependent dissociation constants:

$$k_{d,Na_s^+} = k_{d,Na_s^+}^0 \exp\left\{\frac{(1+\Delta)F\, V_m^{ba}}{3\,R\,T}\right\}$$

$$k_{d,Na_i^+} = k_{d,Na_i^+}^0 \exp\left\{\frac{\Delta\, F\, V_m^{ba}}{3\,R\,T}\right\}$$

Free inorganic phosphate is given by:

$$[Pi] = \frac{[\Sigma Pi]_i}{1 + [K^+]_i/k_{d,K^+Pi} \ + [H^+]_i/k_{d,H^+Pi} \ + [Na^+]_i/k_{d,Na^+Pi}}$$

The term Σ in the denominator of $V_{NaK}(t)$ is given by a sum of permutations of the rate constants

$$\Sigma = \alpha_1^-\alpha_2^-\alpha_3^- + \alpha_1^-\alpha_2^-\alpha_4^+ + \alpha_1^-\alpha_3^+\alpha_4^+ + \alpha_2^+\alpha_3^+\alpha_4^+$$

$$+ \alpha_2^-\alpha_3^-\alpha_4^- + \alpha_1^+\alpha_2^-\alpha_3^- + \alpha_1^+\alpha_2^-\alpha_4^+ + \alpha_1^+\alpha_3^+\alpha_4^+$$

$$+\alpha_1^-\alpha_3^-\alpha_4^- + \alpha_2^+\alpha_3^-\alpha_4^- + \alpha_1^+\alpha_2^+\alpha_3^- + \alpha_1^+\alpha_2^+\alpha_3^+$$

$$+\alpha_1^-\alpha_2^-\alpha_4^- + \alpha_1^-\alpha_3^+\alpha_4^- + \alpha_2^+\alpha_3^+\alpha_4^- + \alpha_1^+\alpha_2^+\alpha_3^+$$

Rate constants used in the pump model are $k_1^+ = 1050\text{s}^{-1}$, $k_1^- = 172.1\text{s}^{-1}\text{mM}^{-1}$, $k_2^+ = 481\text{s}^{-1}$, $k_2^- = 40.1\text{s}^{-1}$, $k_3^+ = 2000\text{s}^{-1}$, $k_3^- = 79{,}287.1\text{s}^{-1}\text{mM}^{-2}$, $k_4^+ = 320\ \text{s}^{-1}$, $k_4^{-1} = 40.1\text{s}^{-1}$. Dissociation constants used are $k_{d,Na_s^+}^0 = 15.5\text{mM}$, $k_{d,Na_i^+}^0 = 2.49\text{mM}$, $k_{d,Na_s^+} = 0.213\text{mM}$, $k_{d,\,K_i^+} = 0.5\text{mM}$, $k_{d,MgATP} = 2.51\text{mM}$, $k_{d,K^+Pi} = 292\text{mM}$, $k_{d,H^+Pi} = 1.69 \times 10^{-4}\ \text{mM}$, and $k_{d,Na^+Pi} = 224\text{mM}$. Other parameters fixed in this model are $[\Sigma Pi]_i = 4.2\text{mM}$, $[H^+]_i = 8 \times 10^{-5}\text{mM}$, $[MgATP] = 9.8\text{mM}$, $[MgADP] = 0.01\text{mM}$, and $\Delta = -0.031$.

Determining paracellular permeability from shunt resistance

In our model we assume that under resting conditions the paracellular pathway is only permeable to Na^+, K^+ and Cl^- ions, therefore the total paracellular or "shunt" current will be given by

$$I_s = I_{Na^+}^{pa} + I_{Cl^-}^{pa} + I_{K^+}^{pa}$$

Assuming the paracellular currents are accurately described by the GHK formulism (equations S5-S7), and that the cation/anion permeability ratio is $1/\lambda$, then in the limit of identical bathing solutions this expression reduces to

$$I_s = P_{pa} \frac{V_t\, \gamma\, F^2}{RT} \left([Na^+] + [K^+] + \lambda\, [Cl^-]\right)$$

where $P_{pa} \equiv P^{pa}_{Na^+} \equiv P^{pa}_{K^+}$ is the permeability of the pathway to cations.

Shunt resistance R_s is related to shunt current via $I_s = V_t/R_s$ therefore

$$P_{pa} = \frac{1/R_s}{(F^2\gamma/RT)\left([Na^+] + [K^+] + \lambda\, [Cl^-]\right)}$$

Mean shunt resistance was measured (Willumsen & Boucher, 1989) to be $412\ \Omega\ cm^2$ and $623\ \Omega\ cm^2$ for non-CF and CF epithelia respectively, with solution composition $[Na^+] = 140mM$, $[Cl^-] = 120mM$, and $[K^+] = 5.2mM$. For a non-selective pathway $\lambda = 1$, we find that

$$P^{non-CF}_{pa} = 3.29074 \times 10^{-8}\ m/s = 0.0329\ \mu m/s$$

$$P^{CF}_{pa} = 2.17616 \times 10^{-8}\ m/s = 0.0218\ \mu m/s$$

If we assume a cation selective paracellular pathway with $\lambda = 1/1.3$ (Levin *et al.*, 2006; Flynn *et al.*, 2009) the values we find are

$$P^{non-CF}_{pa} = 3.67442 \times 10^{-8}\ m/s = 0.0367 \mu m/s$$

$$P^{CF}_{pa} = 2.42989 \times 10^{-8}\ m/s = 0.0243\ \mu m/s$$

All parameter estimation and Monte Carlo filtering analyses were carried out with the following paracellular transport configurations: (a) P_{pa} unchanged in CF and non-selective, (b) P_{pa} reduced in CF relative to non-CF and non-selective, (c) P_{pa} unchanged in CF and cation-selective, (d) P_{pa} reduced in CF relative to non-CF and cation selective.

For simulations where we assumed a cation selective P_{pa} , we also assumed that $P_{Cl^-}^{pa} = 1.4\, P_{gluc^-}^{pa}$. We estimated the ratio of 1.4 based on recreating the experiments of Coakley *et al.* who found a permeability ratio $P_{Cl^-}^{pa} > P_{gluc^-}^{pa}$ (Coakley *et al.*, 2003).

S2 Model validation

We carried out model validation to assess the stability of the dynamical system to small perturbations of model variables. The perturbations were introduced for numerical validation only, and not with the purpose of simulating a particular physiological phenomenon.

Model validation was implemented by adding a forcing term $u(t)$ in a given ODE for a fixed time interval $T1 \to T2$ (one ODE at a time), and analysing if the system remained stable and behaved in a physiologically realistic manner throughout the duration of this interval. The amplitude of the forcing term, u_0, was chosen separately for each variable, to reflect the relevant magnitudes of rates of change that were likely to be seen for that variable during an *in silico* experiment. We found that with the optimal non-CF parameter values, the model ODE's remains stable during and after perturbations of model variables, regardless of the variable (ODE) being perturbed and regardless of whether the forcing term is constant or sinusoidal. Further details of a sample of two of these exercises are provided below.

(a) *Constant input:* we add the forcing term $u(t)$ in the ODE for volume W_i, with $u_0 = 0.1\mu\text{m/s}$, $T1 = 300\text{s}$ & $T2 = 1200\text{s}$, such that

$$u(t) = \begin{cases} 0, & t < T1 \; and \; t > T2 \\ u_o, & T1 \le t \le T2 \end{cases}$$

This modifies the ODE in question as follows

$$\frac{dW_i}{dt} = J_w^{ba}(t) - J_w^{ap}(t) + u(t)$$

The effect of this perturbation on cellular variables such as concentrations and membrane potentials can be seen in Figure S1. The system remains stable during the forcing, and relaxes to the same steady state as it was initially in, after the input term is switched off.

(b) *Sinusoidal input:* we implemented a sinusoidal input into the ODE for moles of Na^+, with $u_0 = 10 \times 10^{-10}\text{mol/cm}^2, \omega = \pi/75\,\text{s}^{-1},\; T1 = 300\text{s}$ & $T2 = 1200\text{s}$

$$u(t) = \begin{cases} 0, & t < T1 \; and \; t > T2 \\ u_o \sin(\omega t), & T1 \le t \le T2 \end{cases}$$

$$\frac{dNa^+}{dt} = J_{NKCC}(t) - 3\, J_{NaK}(t) - I^{ap}_{Na^+}(t)/F\, z_{Na^+} + u(t)$$

See Figure S2 for plots of physiological quantities over time for this perturbation.

S3 Numerical parameter estimation

The problem of estimating model parameters $P_i = \{P^{ap}_{Na^+},\ P^{ap}_{Cl^-},\ P^{ba}_{K^+},\ \rho_{NaK}\ ,\ \rho_{NKCC},\ P^{ba}_{Cl^-}\}$ from observed experimental data $\hat{x} = \left\{\widehat{[Na^+]_\iota}\, , \widehat{[Cl^-]_\iota}\, , \widehat{V^{ap}_m}, \widehat{V_t}\right\}$ can be formulated formally as the following optimisation problem:

$$\min_P \sum_n \left(\left(\frac{\widehat{[Na^+]_\iota}(t_n) - \frac{Na_i^+(t_n)}{W(t_n)}}{\sigma_{\widehat{[Na^+]_\iota}}(t_n)} \right)^2 + \left(\frac{\widehat{[Cl^-]_\iota}(t_n) - \frac{Cl_i^-(t_n)}{W(t_n)}}{\sigma_{\widehat{[Cl^-]_\iota}}(t_n)} \right)^2 + \left(\frac{\widehat{V^{ap}_m}(t_n) - V^{ap}_m(t_n)}{\sigma_{\widehat{V^{ap}_m}}(t_n)} \right)^2 + \left(\frac{\widehat{V_t}(t_n) - V_t(t_n)}{\sigma_{\widehat{V_t}}(t_n)} \right)^2 \right) \tag{S19}$$

subject to the conditions:

1 Model equations: $d\boldsymbol{x}/dt = f(\boldsymbol{x}, \boldsymbol{P})$

2 Initial conditions: $\boldsymbol{x}(t = 0, \boldsymbol{P}) = \boldsymbol{x_0}$

Here t_n are the time points for which experimental data is available, $\hat{x}(t_n)$ is the observed data at that time point, and $x(t_n)$ are the variable values predicted by the model at those time points. $\sigma_{\hat{x}}(t_n)$ is the uncertainty in the experimental data measured at t_n.

The first parameter estimation problem we solved was to find P_{non-CF} which minimised the residuals between observed data from non-CF HNE cells and model output, for a combined data set from two experiments, "*+amiloride* " and "$+0[Cl^-]_\iota$". These *in silico* experiments can be simulated by setting up the model equations and initial conditions appropriately. Amiloride block of ENaC channels is simulated assuming $P^{ap}_{Na^+} = 0$ in model equations $dx/dt = f(x, \boldsymbol{P})$, but with initial conditions x_0 found by solving $f(x, P) = 0$ where $P^{ap}_{Na} \neq 0$. The assumption that this inhibitor only affects the apical Na+ transport pathway is commonly made in modelling studies (Horisberger, 2003;

Falkenberg & Jakobsson, 2010; Garcia *et al.*, 2013) and also is the basis for interpretation of amiloride sensitive V_t measurements in the standard nasal PD test (Knowles *et al.*, 1995).

For modelling how reducing luminal $[Cl^-]$ affects system kinetics, initial conditions are found which solve $f(x, P) = 0$ with $[Cl^-]_l = 120\text{mM}$, and model equations then are numerically integrated from this initial condition with $[Cl^-]_l$ now at 3mM and $[gluconate]$ = 117mM (note we assume that a gluconate concentration is introduced to replace the osmolarity of the Cl^- ions which are removed in this experiment). In both cases the change in model variables x due to the "*+amiloride*" or "$+0[Cl^-]_l$" perturbation can be found by taking the difference between the initial conditions and the new steady state values, $\Delta x_i = x_i(t \gg 0) - (x_0)_i$. The data used along with the resulting model output is shown in Table S3.

The second, separate parameter estimation problem was to find P_{CF} which would minimise residuals between observed data from CF HNE cells and model predicted variable values, again for data from a combination of "*+amiloride*" or "$+0[Cl^-]_l$". The data used in this problem is listed in Table S4.

In order to implement this formal parameter estimation we created a `multistart` optimisation problem in Matlab. A sample of $n = 10^3$ randomly generated sets of parameter values P_i were chosen from a uniform distribution $U(0, 5\,P_b)$ (where P_b is the set of baseline parameter values described in Table 1 of the main text). These were used as start points for multiple runs of the minimisation algorithm. The estimation problem (equation S19) was then solved, using `fmincon` with the `interior point` algorithm, for each randomly chosen start point. The solution which gave the lowest objective function value, out of resulting sample of minima found by the solver, was considered to be the global minimum in the region of parameter space searched and hence the solution to our problem.

The multi-start optimisation problem was run in four different scenarios, to assess the influence of paracellular permeability (P_{pa}) and selectivity ratios ($P^{pa}_{Na^+}/P^{pa}_{Cl^-}$ and $P^{pa}_{Cl^-}/P^{pa}_{gluc^-}$) on the estimated

values of $P^{ap}_{Na^+}$ and $P^{ap}_{Cl^-}$. Initially, we used our baseline estimate for P_{pa} and assumed the paracellular pathway was equally selective (results in Table S5(a)), then we assessed the influence of an increased shunt resistance in CF, again without selectivity of the paracellular pathway (Table S5(b)). The effect of a selective paracellular permeability was then assessed with the baseline value of P_{pa} in both CF and non-CF simulations (Table S6(a)), and the effect of selectivity combined with different CF and non-CF P_{pa} (Table S6(b)).

To determine approximate confidence intervals for the parameter values estimated in this manner, we analysed the remaining parameter sets which gave values of $\chi^2(P)$ within 10% of the global minimum value $\chi^2_{min}(P_{opt})$. Then, for each parameter, we computed the difference between the 90th and 10th percentile of values which resulted in this set of $\chi^2(P)$ values, and used this as guide to the confidence in the original parameter estimate. For example, in Table S6 (b), the value estimated for $P^{ap}_{Na^+}$ is $0.0214\mu m/s$ and only a 2.4% change in this value ($\pm 0.0006\mu m/s$) will increase the sum of squared residuals by up to 10%, where as we can change our estimate of $P^{ba}_{K^+}$ by 82% and only get similar increases in error, implying the estimate of $P^{ap}_{Na^+}$ is significantly better constrained by the data. The second benefit of this confidence interval estimate is that it allows us to make a judgement on whether or not our CF versus non-CF estimates are significantly different, given the data.. Again in Table S6 (b), we can see the CF estimate of $P^{ap}_{Na^+}$ is $\sim 85\%$ greater than the non-CF estimate, but this is much greater than the likely error in both individual estimates, which is $\sim 2\%$ in both cases. Hence this increase is significant given the different sets of HNE cell data.

Figure S3 shows a plot of the model kinetics given by the optimal parameter values we determined in the baseline case, estimated from CF HNE cell data. The results of solving S19 for data from non-CF HNE cells, can be seen in figure 2 of the main text for comparison.

S4 Determining feasible non-CF and CF parameter distributions

Monte Carlo sampling of parameter values from baseline estimates

To generate a population of individual parameter sets (a unique set of parameter values $P_i = \{P^{ap}_{Na^+},\ P^{ap}_{Cl^-},\ P^{ba}_{K^+},\ \rho_{NaK},\ \rho_{NKCC},\ P^{ba}_{Cl^-}\}$) representative of the region of parameter space around the baseline parameter set P_b, individual P_i were randomly chosen from a uniform distribution $(0, 5P_b)$. Each element of P_i was chosen from its own uniform distribution (e.g. $P^{ap}_{Na^+}$ from $(0, 5 \times 0.028\mu\mathrm{m/s})$ etc), and each element was chosen independently of all the other elements. The factor of 5 is arbitrary, this range is an attempt to represent the variability in transport parameters likely to be found in different cultures and cells, by not overlooking any physiologically relevant area of parameter space.

Physiologically realistic steady state behaviour

Equations S13-S18 describe a set of coupled ordinary differential equations (ODE's) of the form $dx/dt = f(x, P)$, where P is the vector of transport parameters as described previously, and $x = \{W_i, Na_i^+, Cl_i^-, K_i^+, V_m^{ap}, V_m^{ba}\}$ is a vector containing the model variables. For the model to predict steady state or homeostatic behaviour, physiological variables must not be changing in time, and hence the condition $dx/dt = 0$ must be satisfied. For a given set of parameter values P_i, we can therefore find the associated steady state variable values x_i by numerically solving the relevant set of non-linear equations, $f(x, P_i) = 0$, for x_i .

In this study we generated $n = 10^6$ parameter sets P_i , and solved $f(x, P_i) = 0$ for each, to find the corresponding set of steady state variable values x_i . The non-linear equations were solved using the Matlab function `fmincon` with the `sqp` algorithm. Parameter sets which predicted un-physiological steady states were discarded. A parameter set was deemed to be un-physiological if it predicted steady state variable values x_i that were not within the ranges which have been observed in HNE cells *in vitro.* Table 2 in the main text contains the upper and lower bounds determined for feasible steady state and kinetic bioelectric properties of normal and CF HNE cells.

We chose the upper and lower bounds on allowed cellular variables to be the 90th and 10th percentile respectively, of the distribution measured for each variable, wherever an appropriate distribution had been published. A distribution of steady state cellular variables has been published for $[Na^{+}], [Cl^{-}], V_m^{ap}$, V_m^{ba} & V_t in normal and CF HNE cells (Willumsen *et al.*, 1989*a*, 1989*b*; Willumsen & Boucher, 1991*b*, 1991*a*).

The upper and lower bounds on the allowed change in cellular variables due to a "+amiloride" or "$+0[Cl^{-}]_l$" experiment was determined differently, as distributions of these quantities was not presented for $in\ vitro$ measurements. Here data for initial and final variable values (say V_t before and after amiloride addition) was published as the mean value of the quantity in question, μ, plus or minus the standard error σ. In order to calculate the relevant upper and lower bounds on $\Delta\mu$, we used $(\mu_{final} - \mu_{initial}) \pm \sqrt{\sigma_{initial}^2 + \sigma_{final}^2}$ since $\sigma_c^2 = \sigma_a^2 + \sigma_b^2$ for the variance in c, when $c = a + b$, and σ_a, σ_b are the variances associated with measurements of a & b respectively.

Physiologically realistic transport kinetics

The second stage of model verification was to find parameter sets which predicted realistic transient behaviour of physiological variables, as well as realistic homeostatic behaviour. To do this we simulated a number of biologically relevant *in silico* experiments, for which experimental data on membrane potential and intracellular concentration kinetics was available.

The two types of simulated experiment carried out were (i) pharmacological block of ENaC channels and (ii) changing luminal Cl^- concentration, and these were implemented by appropriate choice of initial conditions and model equations, as follows:

i. *Amiloride block of ENaC channels.*

Initial conditions are chosen which give a steady state $f(x, P) = 0$ with a non zero ENaC permeability (i.e. $P_{Na^+}^{ap} \neq 0$). With these initial conditions, the system $dx/dt = f(x, P)$ is numerically integrated assuming the ENaC current is completely blocked by amiloride, that is we set $P_{Na^+}^{ap} = 0$ in the model equations.

ii. Reducing [Cl⁻] in the luminal solution.

Initial conditions are chosen which give a steady state $f(x, P) = 0$ when $[Cl^-]_l = 120\text{mM}$. Model equations are numerically integrated, from this initial condition, with $[Cl^-]_l$ now set to 3mM and a gluconate concentration replacing the lost $[Cl^-]_l$ in the luminal compartment $[gluconate]_l = 117\text{mM}$. We assume gluconate cannot permeate through the cell membrane, but will diffuse along the paracellular pathway, creating a current I_{gluc}^{pa}.

The net effect of (i) is to block the apical Na^+ current (i.e.$I_{Na^+}^{ap} \to 0$), and the net effect of (ii) is to increase the driving force for Cl^- secretion across the apical membrane (luminal osmolarity remains the same.

For each parameter set P_i which remained after the initial model verification, the two *in silico* experiments as described above were simulated by numerically integrating the set of ODE's S13-S18, with the steady state value x_i for that particular parameterisation used as the initial condition. Numerical integration of the system $dx/dt = f(x, P)$ was carried out in Matlab using the `ode15s` function, for a time interval much greater than the relaxation period of the system (t=3600s), to allow it to reach a new steady state. The changes in physiological variables between old and new steady states, $\Delta x_i = x_i(t = 3600s) - (x_0)_i$ can be recorded and compared with experiment. P_i which predict Δx_i that are not physiologically realistic can be discarded. The expected changes in steady state variables used as filters are listed in Table 2 in the main text.

Parameter sets which predict steady state and kinetic model behaviour in quantitative agreement with observed data from non-CF HNE cells are shown in Figure S4, and those which can satisfy the CF

constraints in Figure S5. Table S7 (non-CF) and Table S8 (CF) contain the 1st , 25th, 50th, 75th and 99th percentile points of each of these parameter value distributions, so differences between states can be examined. Focusing on the non-CF parameter distributions, multiple combinations of transport parameter values can explain the observed bioelectric properties, but the model equations, in combination with the bounds placed on model output, impose a structure on the acceptable region of parameter space. Several correlations between parameter values can be seen (ρ_{NaK} & $P_{K^+}^{ba}$ for example), and several transport parameters can only assume values within a bounded region of the parameter space searched ($P_{Na^+}^{ap}$& $P_{Cl^-}^{ap}$). It is clear that the value of other transport parameters, ρ_{NKCC} for example, have not been constrained. Any value of ρ_{NKCC} within the region searched for this parameter would allow the observed data to be reproduced, so long as the other parameter values were chosen appropriately.

Influence of paracellular permeability & selectivity on feasible parameter distributions

As well as performing the MC filtering analysis for our baseline estimate value of P_{pa} , we also repeated the analysis to assess whether increased shunt resistance in CF or selective paracellular permeability would significantly alter the outcomes. In line with our parameter estimation work, we separately investigated the effect of (i) reduced P_{pa} in CF HNE cells, (ii) differential ionic selectivity in P_{pa} and (iii) reduced P_{pa} in CF combined with selective paracellular transport. The results of these further MC filtering analysis can be seen in figures Figure S6 - Figure S8. Qualitatively the relative difference between CF and non-CF $P_{Na^+}^{ap}$ & $P_{Cl^-}^{ap}$ distributions is not altered in either of these scenarios, although the estimates of $P_{Cl^-}^{ap}$ values are decreased if we assume $P_{Cl^-}^{pa}/P_{gluc^-}^{pa} > 1$.

S5 Model sensitivity analysis

To quantitatively assess the relative influence of transport parameters on physiological variables of interest, we carried out a sensitivity analysis using the data resulting from the model verification process. The approach we take is similar to that of Taylor, Goaillard and Marder (Taylor *et al.*, 2009) where the authors are determining how different conductances affect electrophysiological properties of an multi-compartmental cellular model of a neuron, and Sobie (Sobie, 2009), in the case of analysing cardiac myocyte models.

The data remaining from the Monte Carlo filtering analysis consists of a sample of physiologically feasible parameter sets $P_i = \{P^{ap}_{Na^+},\ P^{ap}_{Cl^-},\ P^{ba}_{K^+},\ \rho_{NaK}\ ,\ \rho_{NKCC},\ P^{ba}_{Cl^-}\}$ and steady state and kinetic model outputs $y = \{x_i, \Delta x_i\}$. For a given physiological quantity of interest, predicted by the model, we fit a multiple regression model between input parameter sets P_i and their predicted value of that quantity y:

$$y = b_0 + \sum_{i=1}^{n} \left(b_i P_i + b_{ii} P_i^2\right) + \sum_{i=1}^{n} \sum_{j=i+1}^{n} b_{ij} P_i P_j \qquad \text{(S20)}$$

$$y = \{V_t, "\Delta V_t + amiloride", "\Delta V_t + 0[Cl^-]_l"\} \qquad \text{(S21)}$$

$$P_i = \{P^{ap}_{Na^+},\ P^{ap}_{Cl^-},\ P^{ba}_{K^+},\ \rho_{NaK}\ ,\ \rho_{NKCC},\ P^{ba}_{Cl^-}\} \qquad \text{(S22)}$$

Before fitting the regression model, parameter values are z-scored. That is, the mean and standard deviation of the remaining sample of values of an individual transport parameter, say $P^{ap}_{Cl^-}$, is calculated. Then each value in the remaining population of $P^{ap}_{Cl^-}$ is reduced by the mean, and normalised by the standard deviation. Doing this ensures that the range of values remaining for each parameter are on a similar scale.

Once the population of parameter sets is z-scored, the regression model was fit using Matlab function `regstats` with option `'quadratic'`. The regression coefficients b_i determine the strength of the linear correlation between parameter P_i and output y. By comparing the magnitude

of the linear regression coefficients we have a way of objectively determining the relative influence that transport parameters have on the physiological variable of interest. In our study we carry out sensitivity analyses to determine the relationships between transport parameters and basal V_t, $\Delta V_t + amiloride$, and $\Delta V_t + 0[Cl^-]_l$. The results of these analyses are listed in Table S9.

S6 References

Benjamin BA & Johnson EA (1997). A quantitative description of the Na-K-2Cl cotransporter and its conformity to experimental data. *American journal of physiology Renal physiology* **273,** F473–482.

Coakley RD, Grubb BR, Paradiso AM, Gatzy JT, Johnson LG, Kreda SM, O'Neal WK & Boucher RC (2003). Abnormal surface liquid pH regulation by cultured cystic fibrosis bronchial epithelium. *Proceedings of the National Academy of Sciences of the United States of America* **100,** 16083–16088.

Falkenberg C V & Jakobsson E (2010). A biophysical model for integration of electrical, osmotic, and pH regulation in the human bronchial epithelium. *Biophysical journal* **98,** 1476–1485.

Flynn AN, Itani O a, Moninger TO & Welsh MJ (2009). Acute regulation of tight junction ion selectivity in human airway epithelia. *Proceedings of the National Academy of Sciences of the United States of America* **106,** 3591–3596.

Garcia GJM, Boucher RC & Elston TC (2013). Biophysical Model of Ion Transport across Human Respiratory Epithelia Allows Quantification of Ion Permeabilities. *Biophysical Journal* **104,** 716–726.

Hille B (2001). *Ion channels of excitable membranes*. Sinauer.

Horisberger J-D (2003). ENaC-CFTR interactions: the role of electrical coupling of ion fluxes explored in an epithelial cell model. *Pflügers Archiv European Journal of Physiology* **445,** 522–528.

Knowles MR, Paradiso AM & Boucher RC (1995). In vivo nasal potential difference: techniques and protocols for assessing efficacy of gene transfer in cystic fibrosis. *Human gene therapy* **6,** 445–455.

Levin MH, Kim JK, Hu J & Verkman AS (2006). Potential difference measurements of ocular surface Na+ absorption analyzed using an electrokinetic model. *Investigative ophthalmology & visual science* **47,** 306–316.

Smith NP & Crampin EJ (2004). Development of models of active ion transport for whole-cell modelling: cardiac sodium-potassium pump as a case study. *Progress in biophysics and molecular biology* **85,** 387–405.

Sobie EA (2009). Parameter sensitivity analysis in electrophysiological models using multivariable regression. *Biophysical journal* **96,** 1264–1274.

Taylor AL, Goaillard J-M & Marder E (2009). How multiple conductances determine electrophysiological properties in a multicompartment model. *The Journal of neuroscience : the official journal of the Society for Neuroscience* **29,** 5573–5586.

Warren NJ, Tawhai MH & Crampin EJ (2009). A mathematical model of calcium-induced fluid secretion in airway epithelium. *Journal of theoretical biology* **259,** 837–849.

Willumsen NJ & Boucher RC (1989). Shunt resistance and ion permeabilities in normal and cystic fibrosis airway epithelia. *American Journal Of Physiology* **256,** C1054–63.

Willumsen NJ & Boucher RC (1991*b*). Transcellular sodium transport in cultured cystic fibrosis human nasal epithelium. *American journal of physiology Cell physiology* **261,** C332–341.

Willumsen NJ & Boucher RC (1991*a*). Sodium transport and intracellular sodium activity in cultured human nasal epithelium. *American Journal Of Physiology* **261,** C319–31.

Willumsen NJ, Davis CW & Boucher RC (1989*a*). Intracellular Cl- activity and cellular Cl- pathways in cultured human airway epithelium. *American Journal Of Physiology* **256,** C1033–44.

Willumsen NJ, Davis CW & Boucher RC (1989*b*). Cellular Cl- transport in cultured cystic fibrosis airway epithelium. *American Journal Of Physiology* **256,** C1045–53.

S7 Tables

Quantity	Symbol	Units	Subscript / Superscript
Time	t	s	-
Volume	W	μm^3	-
Water flux	J_w^q	$\mu m^3/s$	$q = \{ap, ba\}$
Pump/co-transporter flux	J_q	10^{-10} mol/cm^2	$q = \{NaK, NKCC\}$
Ion current	I_n^x	$\mu A/cm^2$	$n = \{Na^+, Cl^-, K^+, gluc\}$, $x = \{ap, ba, pa\}$
Permeability	P_n^x	μm/s	$n = \{Na^+, Cl^-, K^+, gluc\}$, $x = \{ap, ba, pa\}$
Thermodynamic activity	a_q^n	mM	$q = \{i, l, m\}$, $n = \{Na^+, Cl^-, K^+, gluc\}$
Activity coefficient	γ	-	-
Valence	z_n	-	$n = \{Na^+, Cl^-, K^+, gluc^-\}$
Membrane potential	V_m^x	mV	$x = \{ap, ba\}$
Trans-epithelial PD	V_t	mV	-
Hydraulic conductivity	L_w	μm/s	-
Partial molar volume	v_w	m^3/mol	-
Impermeable anions	ψ_i	mol	-
Osmolarity	$[S]_q$	mOsm/L	$q = \{l, s\}$
Ionic concentration	$[n]_i$	mM	$n = \{Na^+, Cl^-, K^+, gluc\}$
Moles of ions	$n_i^{+/-}$	mol	$n = \{Na^+, Cl^-, K^+, gluc\}$
Membrane capacitance	C_m	$\mu F/cm^2$	-
Faraday constant	F	C/mol	-
Universal gas constant	R	J/mol K	-
Temperature	T	K	-

Table S1: Notation used in mathematical model. Subscripts/superscript abbreviations: i – intracellular, l – luminal, s – serosal, ap – apical, ba – basolateral, pa - paracellular,

Parameter	Value	Units	Source
P_{pa}	0.035	$\mu m/s$	(Willumsen & Boucher, 1989)
γ	0.77	-	(Hille, 2001)
L_w	220	$\mu m/s$	(Falkenberg & Jakobsson, 2010)
v_w	1.805×10^{-5}	m^3/mol	(Warren *et al.*, 2009)
ψ_i	2.375×10^{-15}	mol	-
$[S]_{l,s}$	290	mOsm/L	-
C_m	1	$\mu F/cm^2$	(Hille, 2001)
F	96485	C/mol	(Hille, 2001)
R	8.314	J/mol K	(Hille, 2001)
T	310	K	(Hille, 2001)

Table S2: Fixed parameter values used in mathematical model

Experiment	n	Time (t_n)	(a) Observed data				(b) Model output			
			$\widehat{[Na^+]_\iota}(t_n)$	$\widehat{[Cl^-]_\iota}(t_n)$	$\widehat{V_m^{ap}}(t_n)$	$\widehat{V}_t(t_n)$	$[Na^+]_i(t_n)$	$[Cl^-]_i(t_n)$	$V_m^{ap}(t_n)$	$V_t(t_n)$
$+amiloride$	1	0	26.8 ± 1.9	-	-18.7 ± 1.5	-12.2 ± 2.5	26.9	-	-21.9	-12.5
	2	1050	23.8 ± 1.9	-	-27.3 ± 2.5	-5.1 ± 1.0	21.4	-	-33.7	-5.2
	3	0	-	57.0 ± 3.5	-25.9 ± 2.5	-11.8 ± 1.5	-	50.2	-21.9	-12.5
	4	1050	-	52.2 ± 4.7	-36.6 ± 2.2	-5.0 ± 0.6	-	49.6	-33.7	-5.2
$+0[Cl^-]_l$	5	0	-	55.5 ± 5.2	-22.5 ± 1.4	-12.4 ± 2.4	-	50.2	-21.9	-12.5
	6	1050	-	35.1 ± 4.5	-10.4 ± 2.5	-21.8 ± 2.2	-	47.4	-8.9	-20.1

Table S3: (a) Experimental data used for estimation of *non-CF* transport parameters. Concentrations are given in mM, membrane potentials in mV, and time in seconds. Equivalent data predicted by optimal parameter set in the case of baseline P_{pa} and non-selective paracellular transport are shown in (b).

Sources of observed data:

$\left\{a_i^{Na^+}, V_t, V_m^{ap}\right\}$ for n=1, 2 : Data found by digitizing plots in Figure 8 of reference (Willumsen & Boucher, 1991*a*), using the initial steady state and final data points from each time course plot.

$\left\{a_i^{Cl^-}, V_t, V_m^{ap}\right\}$ for n = 3, 4: Table 4 in (Willumsen *et al.*, 1989*a*)*

$\left\{a_i^{Cl^-}, V_t, V_m^{ap}\right\}$ for n = 5, 6: Table 3 in (Willumsen *et al.*, 1989*a*)*

Experiment	n	Time (t_n)	(a) Observed data				(b) Model output			
			$\widehat{[Na^+]_\iota}(t_n)$	$\widehat{[Cl^-]_\iota}(t_n)$	$\widehat{V_m^{ap}}(t_n)$	$\widehat{V}_t(t_n)$	$[Na^+]_i(t_n$	$[Cl^-]_i(t_n)$	$V_m^{ap}(t_n)$	$V_t(t_n)$
$+amiloride$	1	0	-	67.7 ± 5.6	-8.2 ± 5.0	-37.4 ± 8.4	-	59.1	-13.1	-29.6
	2	1050	-	66.6 ± 6.5	-43.6 ± 4.0	-1.2 ± 1.1	-	63.6	-48.3	-1.6
	3	0	25.8 ± 3.9	-	-7.1 ± 5.0	-42.3 ± 6.0	22.7	-	-13.1	-29.6
	4	1050	40.6 ± 3.9	-	-48.9 ± 2.5	-2.0 ± 1.0	18.1	-	-48.3	-1.6
$+0[Cl^-]_l$	5	0	-	62.7 ± 6.2	-20.1 ± 9.2	-27.7 ± 6.1	-	59.1	-13.1	-29.6
	6	1050	-	65.1 ± 6.4	-17.1 ± 9.5	-31.0 ± 5.5	-	59.0	-12.0	-30.3

Table S4: (a) Experimental data used for estimation of *CF* transport parameters. (b) Equivalent data predicted by optimal parameter set in the case of baseline P_{pa} and non-selective paracellular transport.

Sources of observed data are:

$\{a_i^{Cl^-}, V_m^{ap}, V_t\}$ for n = 1, 2: Table 3 in (Willumsen *et al.*, 1989*b*)*

$\left\{a_i^{Na^+}, V_t, V_m^{ap}\right\}$ for n = 3, 4: Data found by digitizing plots in Figure 7 of reference (Willumsen & Boucher, 1991*b*), using the initial steady state and final data points from each time course plot.

$\left\{a_i^{Cl^-}, V_t, V_m^{ap}\right\}$ for n = 5, 6: Table 2 in (Willumsen *et al.*, 1989*b*)*

* Assuming system is at steady state when measurements are made at $t > 5\text{mins}$.

Note since measurements of V_m^{ap} and V_t from two separate "*+amiloride*" experiments are included in the objective function, we weight the residual error from each of these data points by ½, in order to give the same weight to these measurements as to $[Cl^-]$ and $[Na^+]$, for which only 1 set of measurements is available.

Parameter	(a) Baseline P_{pa} , no selectivity				(b) P_{pa} different in CF, no selectivity			
	Non-CF		CF		Non-CF		CF	
	Estimate	Range	Estimate	Range	Estimate	Range	Estimate	Range
$P^{ap}_{Na^+}$	0.0244	0.0003	0.0650	0.0017	0.0230	0.0002	0.0419	0.0009
$P^{ap}_{Cl^-}$	0.0659	0.0033	0.0057	0.0007	0.0619	0.0033	0.0035	0.0005
$P^{ba}_{K^+}$	0.1026	0.1028	0.4000	0.0653	0.0960	0.1118	0.3429	0.0612
ρ_{NaK}	0.1271	0.0678	0.4894	0.0530	0.1191	0.0733	0.3653	0.0453
ρ_{NKCC}	0.1875	0.5146	2.0000	0.4892	0.1744	0.5122	2.0000	0.5372
$P^{ba}_{Cl^-}$	0.0971	0.1760	0.1442	0.0252	0.0904	0.1937	0.1268	0.0242
$P^{Na^+}_{pa}$	0.0350	-	0.0350	-	0.0329	-	0.0218	-
$P^{Na^+}_{pa} / P^{Cl^-}_{pa}$	1.0	-	1.0	-	1.0	-	1.0	-
$P^{Cl^-}_{pa} / P^{gluc^-}_{pa}$	1.0	-	1.0	-	1.0	-	1.0	-

Table S5: Results of parameter estimation via minimisation of residual error between model predictions and experimental data given in tables S3 and S4. (a) Estimates of transport parameters found when P_{pa} is assumed to be fixed at baseline estimate in both CF and non-CF simulations, and P_{pa} is assumed to be the same for all ions. (b) Parameter estimates when paracellular permeability is assumed to be decreased in CF (Willumsen & Boucher, 1989) **with no selectivity in this pathway.**

Parameter	(a) Baseline P_{pa} , cation selective				(b) Different P_{pa} and cation selective			
	Non-CF		CF		Non-CF		CF	
	Estimate	Range	Estimate	Range	Estimate	Range	Estimate	Range
$P^{ap}_{Na^+}$	0.0205	0.0006	0.0563	0.0020	0.0214	0.0006	0.0398	0.0009
$P^{ap}_{Cl^-}$	0.0466	0.0019	0.0048	0.0007	0.0489	0.0015	0.0033	0.0005
$P^{ba}_{K^+}$	0.0789	0.0621	0.3971	0.0836	0.0823	0.0675	0.3336	0.0624
ρ_{NaK}	0.0983	0.0349	0.4629	0.0713	0.1030	0.0347	0.3520	0.0477
ρ_{NKCC}	0.1477	0.3729	1.9999	0.6230	0.1529	0.4117	2.0000	0.4994
$P^{ba}_{Cl^-}$	0.0604	0.0950	0.1447	0.0329	0.0626	0.1026	0.1236	0.0253
$P^{Na^+}_{pa}$	0.0350	-	0.0350	-	0.0367	-	0.0243	-
$P^{Na^+}_{pa} / P^{Cl^-}_{pa}$	1.3	-	1.3	-	1.3	-	1.3	-
$P^{Cl^-}_{pa} / P^{gluc^-}_{pa}$	1.4	-	1.4	-	1.4	-	1.4	-

Table S6: (a) Estimates of transport parameter values founds when assuming baseline P_{pa} with cation selectivity in the pathway, and an increase in the Cl^- / gluconate$^-$ selectivity ratio. (b) Estimates of transport parameters assuming selective paracellular permeability, and a decreased P_{pa} in CF.

Parameter	Percentile 1st	25th	Median	75th	99th
$P^{ap}_{Na^+}$	0.0131	0.0184	0.0224	0.0268	0.0358
$P^{ap}_{Cl^-}$	0.0458	0.0633	0.0735	0.0846	0.1096
$P^{ba}_{K^+}$	0.0718	0.1866	0.2506	0.3165	0.3950
ρ_{NaK}	0.0460	0.1723	0.3207	0.5018	0.8706
ρ_{NKCC}	0.0825	0.5081	0.9189	1.4250	1.9709
$P^{ba}_{Cl^-}$	0.0814	0.2583	0.3503	0.4267	0.4965

Table S7: Percentile value data for transport parameters, from distributions remaining after non-CF constraints were applied to model outputs (baseline, non-selective P_{pa}).

Parameter	Percentile 1st	25th	Median	75th	99th
$P^{ap}_{Na^+}$	0.0753	0.1030	0.1180	0.1292	0.1395
$P^{ap}_{Cl^-}$	0.0004	0.0090	0.0184	0.0302	0.0707
$P^{ba}_{K^+}$	0.2294	0.3134	0.3504	0.3779	0.3995
ρ_{NaK}	0.0800	0.2232	0.3793	0.5674	0.9625
ρ_{NKCC}	0.0510	0.2718	0.6578	1.2262	1.9650
$P^{ba}_{Cl^-}$	0.0419	0.1648	0.2617	0.3603	0.4912

Table S8: Percentile value data for transport parameters, from distributions remaining after CF constraints were applied to model outputs (baseline, non-selective P_{pa}).

Parameter	Basal V_t (mV)	$\Delta V_t + amiloride$ (mV)	$\Delta V_t + 0\ [Cl^-]_l$ (mV)
$P^{ap}_{Na^+}$	-1.4849	1.4772	-0.0275
$P^{ap}_{Cl^-}$	-0.0202	-0.5660	-1.5210
$P^{ba}_{K^+}$	-1.7234	0.5365	0.6121
ρ_{NaK}	-0.0111	-0.2837	-0.4626
ρ_{NKCC}	0.3135	-0.4531	-0.3959
$P^{ba}_{Cl^-}$	1.1136	-0.0281	-0.4696

Table S9: Linear regression coefficients (b_i) found by fitting multiple regression between z-scored transport parameters ($\widetilde{P_i}$) and physiological variables (y), for use in sensitivity analysis.

S8 Figures

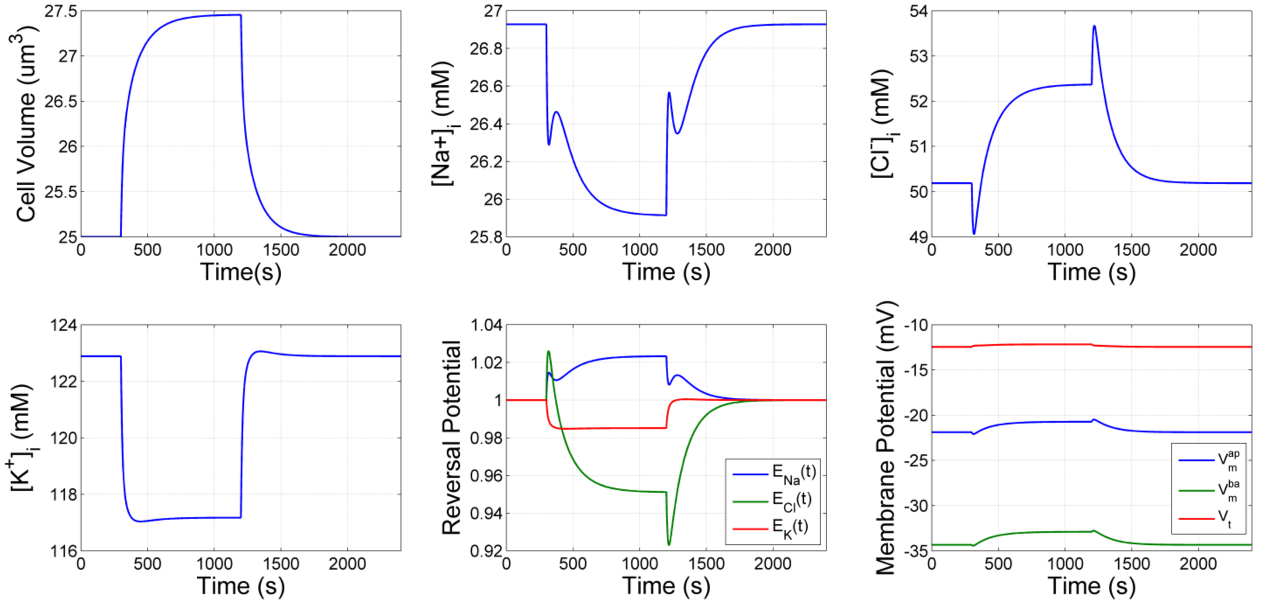

Figure S1: Model validation with constant input. The rate of change of cell volume is altered so that there is a constant forcing input between $T1 = 300s$ and $T2 = 1200s$. Other cellular quantities change accordingly, and the system reaches a new steady state $\sim t = 1000s$, before relaxes back to the initial steady state once the constant input is switched off. Note reversal potentials are normalised to their initial steady state value (Na^+: +44.0 mV, Cl^-: -23.3 mV, K^+: -85.5 mV)

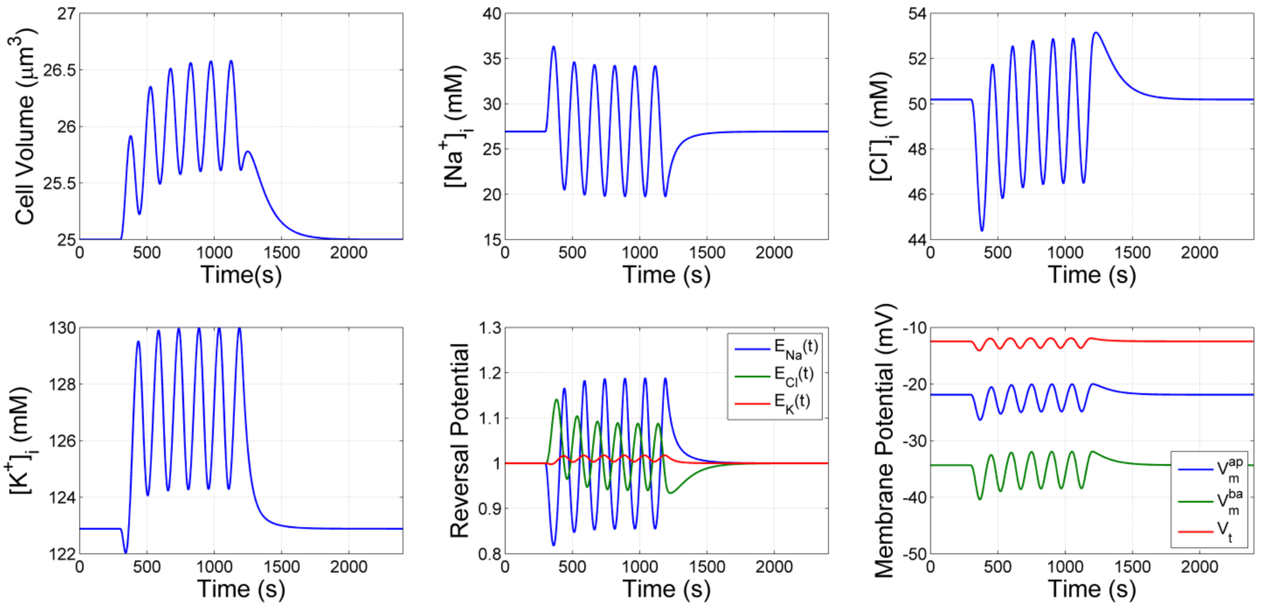

Figure S2: Model validation with sinusoidal input. The ODE for rate of change of Na^+ moles in the cell is perturbed with a sinusoidally varying input term between $T1 = 300s$ and $T2 = 1200s$. All other cellular variables are affected to some extent by this forcing of Na_i^+ and vary with the same periodicity as the input, before returning to the same steady state as they were initially in, after the forcing term is switched off. Note reversal potentials are normalised as in figure S1.

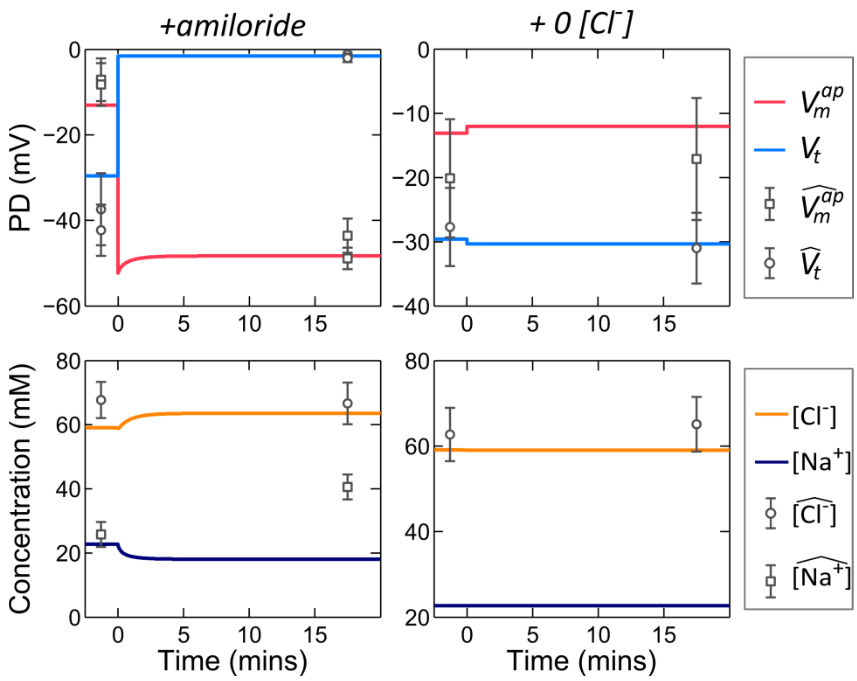

Figure S3: Comparison of model output against observed data for V_m^{ap} (model in red, data points squares) , V_t (model blue, data points circles), $[Na^+]_i$ (model purple, data points square) and $[Cl^-]_i$ (model yellow, data points circles), for two simulations, amiloride addition and removal of luminal Cl$^-$. Parameters used for simulations are those which minimised residual error between data from CF cells and model predictions for the combination of these two experiments (see Table S5(a)).

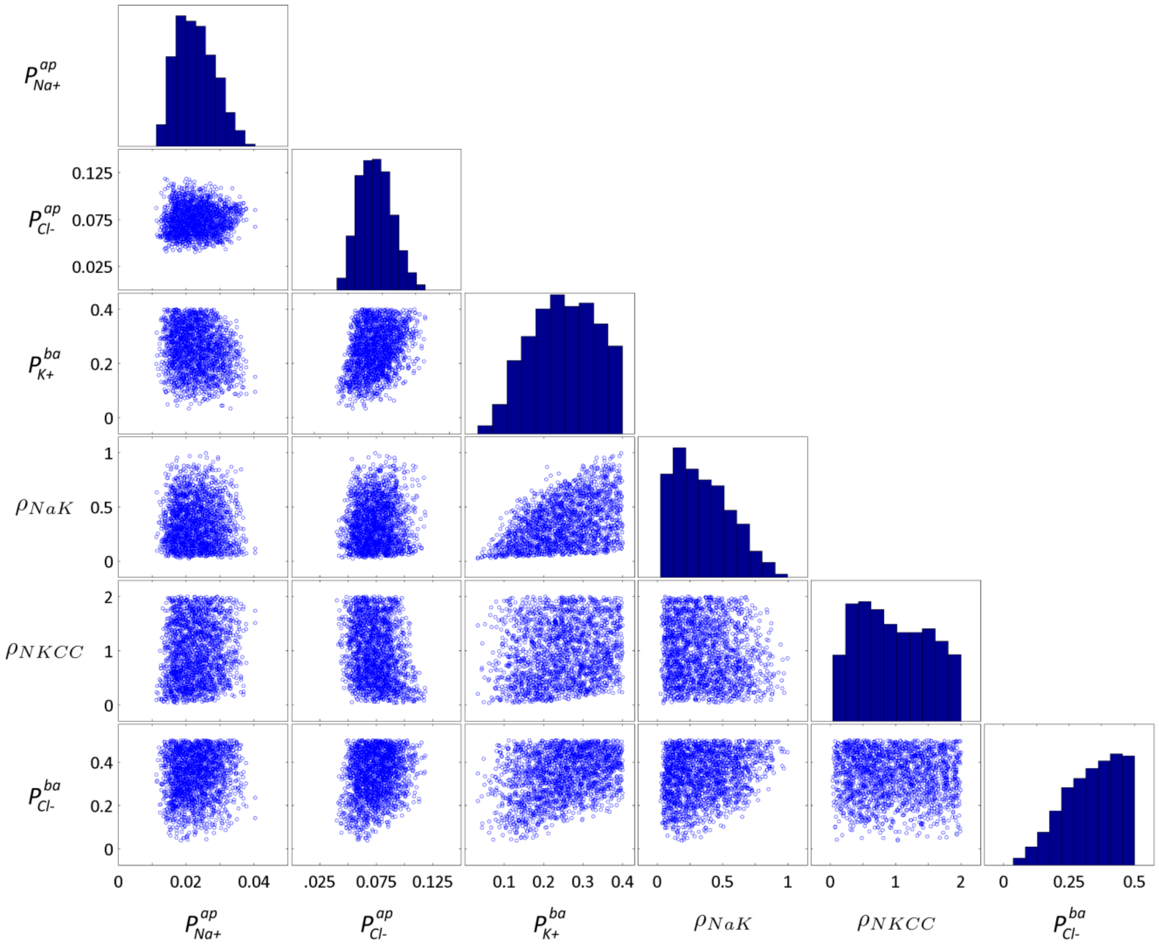

Figure S4: Distributions of parameter values remaining after placing non-CF constraints on our Monte Carlo sample of model simulations. $P^{ap}_{Na^+}$ and $P^{ap}_{Cl^-}$ are constrained, $P^{ba}_{K^+}$ & ρ_{NaK} are correlated, and ρ_{NKCC} & $P^{ba}_{Cl^-}$ are not constrained.

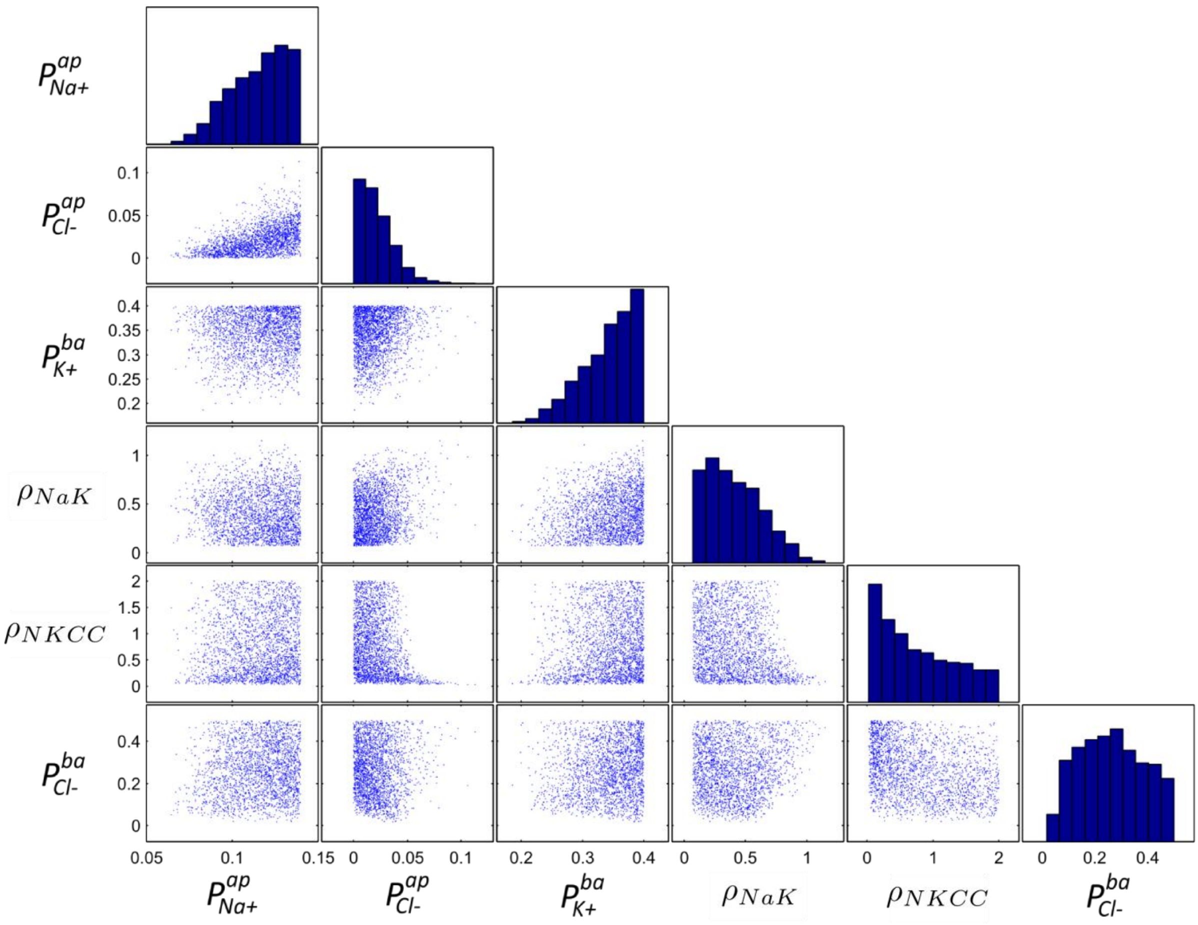

Figure S5: Distributions of parameter values remaining after placing CF constraints on our Monte Carlo sample of model simulations. Distributions found here are significantly different from those found in the non-CF population for some parameters, such as $P^{ap}_{Na^+}$ and $P^{ap}_{Cl^-}$. Others such as ρ_{NKCC} remain un-constrained.

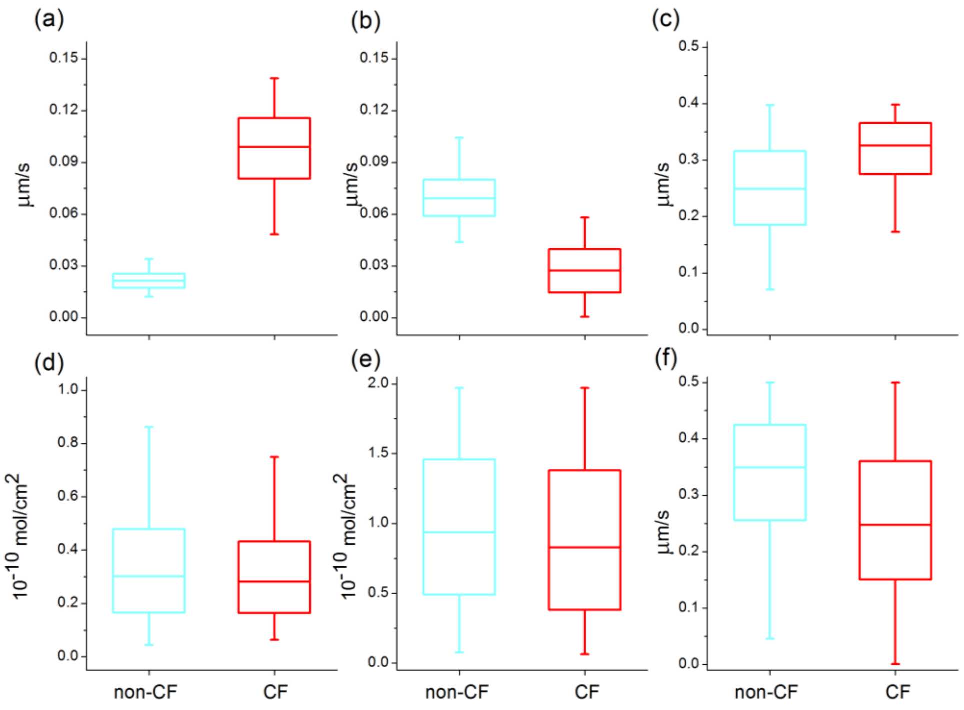

Figure S6: Effect of decreased paracellular permeability P_{pa} in CF on feasible distributions of transport parameter values found via MC filtering analysis. Distributions shown are for (a) $P^{ap}_{Na^+}$, (b) $P^{ap}_{Cl^-}$, (c) $P^{ba}_{K^+}$, (d) ρ_{NaK} , (e) ρ_{NKCC} and (f) $P^{ba}_{Cl^-}$ (cyan: non-CF, red: CF).

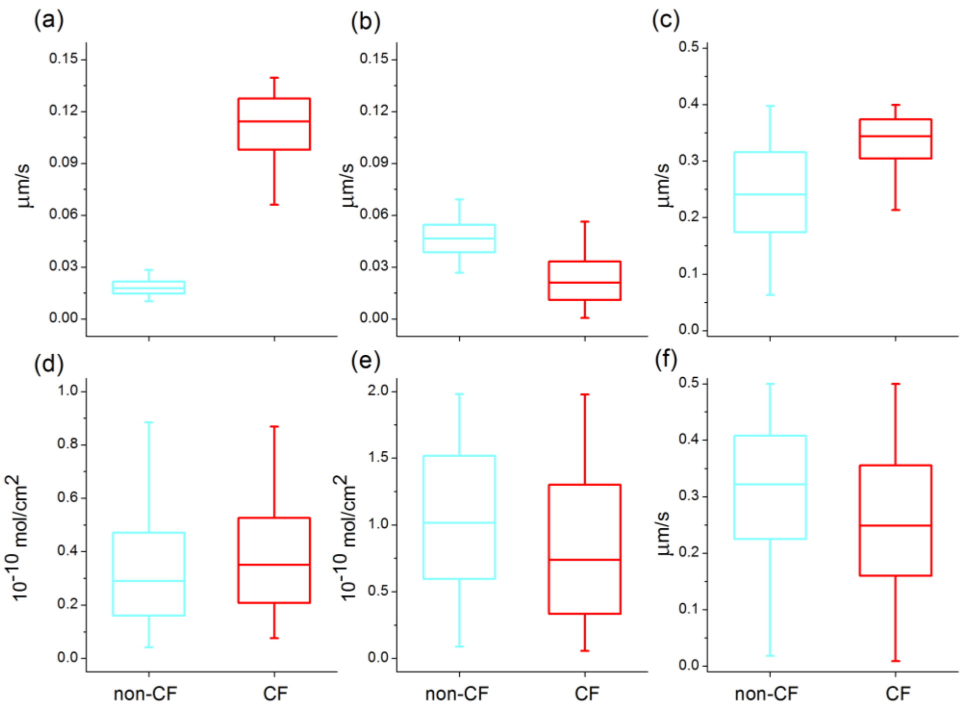

Figure S7: Effect of selective P_{pa} on feasible distributions of transport parameters found via MC filtering analysis. Paracellular pathway is more permeable to cations over anions, and to Cl^- over gluconate. Distributions shown are as in figure S6; (a) $P^{ap}_{Na^+}$, (b) $P^{ap}_{Cl^-}$, (c) $P^{ba}_{K^+}$, (d) ρ_{NaK} , (e) ρ_{NKCC} and (f) $P^{ba}_{Cl^-}$ (cyan: non-CF, red: CF).

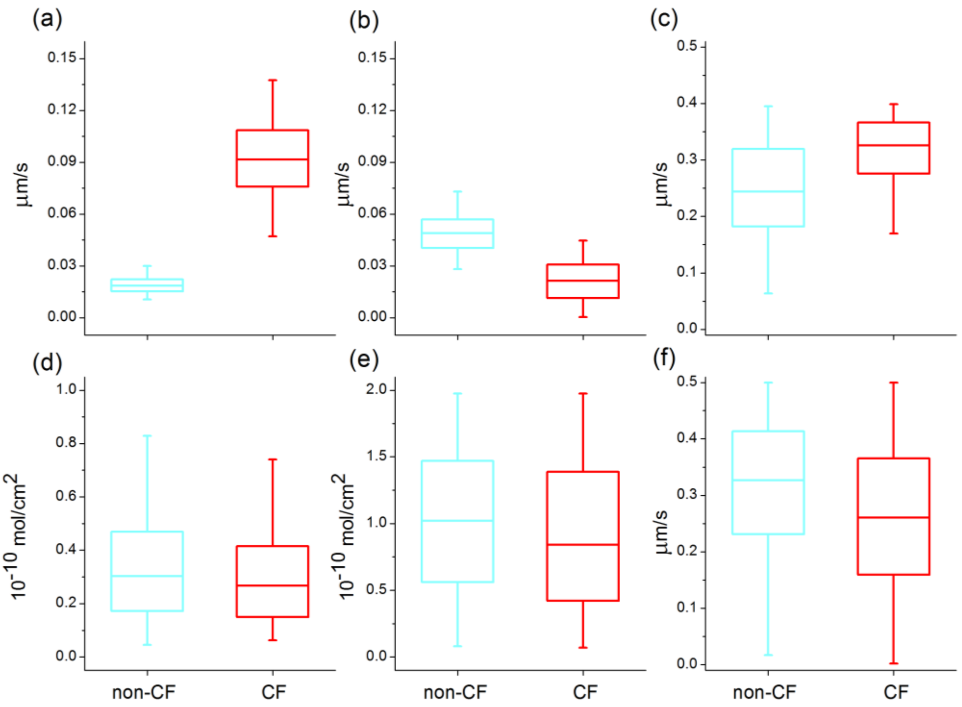

Figure S8: Effect of reduced P_{pa} in CF, combined with selective paracellular transport, on feasible non-CF (cyan) and CF (red) parameter distributions found via MC filtering analysis. Distributions shown are for (a) $P_{Na^+}^{ap}$, (b) $P_{Cl^-}^{ap}$, (c) $P_{K^+}^{ba}$, (d) ρ_{NaK} , (e) ρ_{NKCC} and (f) $P_{Cl^-}^{ba}$.

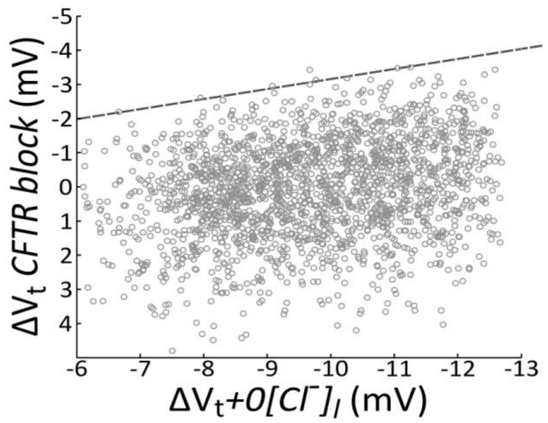

Figure S9: $\Delta V_t + 0[Cl^-]_l$ is commonly used as a proxy measure for $P_{Cl^-}^{ap}$ in airway epithelial cells. Here it is plotted against ΔV_t inducing by blocking CFTR (i.e. $P_{Cl^-}^{ap} \rightarrow 0$), for the distribution of non-CF values found. For a given low Cl⁻ response, hyperpolarisation or depolarisation of basal V_t is possible, but there appears to be a limit on the hyperpolarisation possible (suggested by dashed line), which is significantly less than the magnitude observed in CF.
